# Restoring access to long-term social recognition memories disrupted by sleep deprivation

**DOI:** 10.1126/sciadv.adu9805

**Published:** 2026-06-10

**Authors:** Adithya Sarma, Camilla Paraciani, Junfei Cao, Evgeniya Tyumeneva, Caterina Stacchiola, Elroy L. Meijer, Nienke de Vries, Soraya Smit, Fleur Meijer, Marit Bonne, Jean-Christophe Billeter, Peter Meerlo, Robbert Havekes

**Affiliations:** ^1^ Neurobiology Expertise Group, Groningen Institute for Evolutionary Life Sciences (GELIFES), University of Groningen, Groningen, Netherlands.; ^2^ Evolutionary Genetics, Development & Behaviour Group, Groningen Institute for Evolutionary Life Sciences (GELIFES), University of Groningen, Groningen, Netherlands.

## Abstract

Long-term social memories are vital for forming and maintaining relationships, and social amnesia can disrupt daily life. Using a new paradigm to study these hippocampus-dependent memories, we show that mice can distinguish between multiple social experiences even when they occur in the same context across days. Sleep deprivation immediately after socialization disrupts memory consolidation, leading to social amnesia. Treatment with the Food and Drug Administration–approved phosphodiesterase type 4 inhibitor roflumilast during sleep deprivation protects memory consolidation, while administration immediately before testing reverses social amnesia temporarily. Optogenetic reactivation of dentate gyrus engram cells restores social memory access and enables selective retrieval of individual memories. In addition, using two cFos-based engram-tagging strategies, we find that sleep deprivation selectively affects reactivation of experience-specific engrams while not affecting engram formation or overlap. These results suggest that impaired engram reactivation contributes to sleep deprivation–induced social amnesia and highlights the role of the hippocampal dentate gyrus in maintaining and distinguishing social experiences in a single context.

## Teaser

Sleep deprivation causes social amnesia by blocking access to long-term social memories rather than erasing them.

## INTRODUCTION

Social recognition memory (SRM)—the ability to recognize and remember individual social experiences—is essential for survival in social animals. Given its importance, social memory has been extensively studied across species, revealing its role in enabling animals to form social bonds, foster cooperation, and maintain stable relationships (*1*–*4*). While these studies have advanced our understanding of the mechanisms underlying social memory and its functional importance, there is a lack of understanding how long-term social recognition memories persist over several days. A recent meta-analysis has shown that there is a limited understanding of the neural mechanisms underlying long-term social memories as existing protocols are tailored to investigate social memories across a few hours (*5*). Furthermore, it is often the case that individuals have multiple social experiences over time, necessitating the ability to distinguish these encounters as separate memories. However, this critical aspect of social memory processing has not received attention. Linked to these two concerns, it has been challenging to study how sleep and sleep loss modulate multiple long-term social memories as such studies depend on the memory being naturally retained over several days. With sleep deprivation (SD) becoming increasingly prevalent in modern society, understanding its effects on individual long-term social memories is becoming increasingly critical (*6*, *7*). To address these challenges, we developed an experimental design that (i) enabled the study of social recognition memories across multiple days, (ii) facilitated the investigation of neural circuits underlying multiple social experiences, and (iii) helped delineate whether sleep and sleep loss affect the neurobiological mechanisms underlying long-term social recognition memories.

## RESULTS

### A 6-hour SD period immediately following socialization impairs long-term SRM

**Fig. 1.**
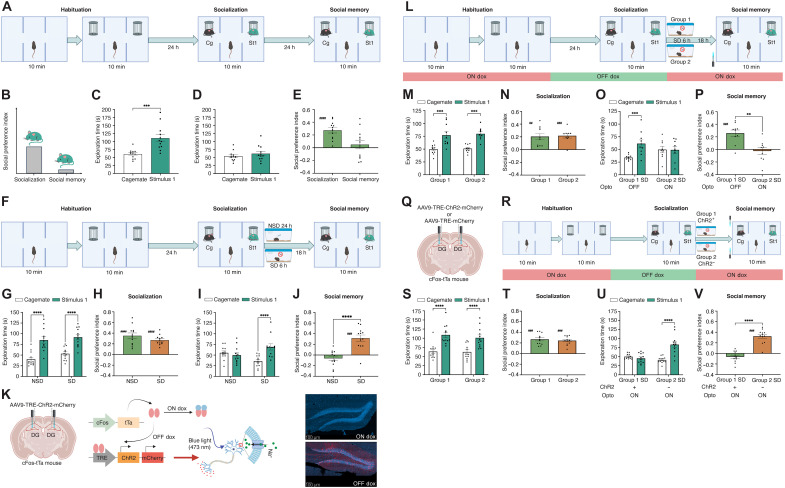
Long-term social recognition memories impaired by SD can be retrieved by optogenetic reactivation of the tagged memory engram. (**A**) Mice were habituated to an arena with two wire cups in opposing chambers. After 24 hours (h), mice were exposed to the cagemate and novel stimulus. Social memory was assessed 24 hours later by reexposing mice to the same animals. (**B**) Socialization: Mice were expected to preferentially explore the novel stimulus, reflected by a positive social preference index (SPI). Social memory (SM): No preference was expected if memory was consolidated. (**C**) Socialization: Mice preferentially explored the novel stimulus. (**D**) SM: Mice explored both animals equally. (**E**) SPI was positive during socialization but not during SM, indicating successful retrieval. (**F**) Mice underwent 6 hours of SD following socialization (SD) or were left undisturbed (NSD). (**G** and **H**) Socialization: Sleep-deprived and non–sleep-deprived mice preferentially explored the stimulus. (**I** and **J**) SM: Non–sleep-deprived mice showed no preference. Sleep-deprived mice preferred the stimulus. (**K**) cFos-tTA mice were injected with AAV9-TRE-ChR2-mCherry and implanted with optical fibers targeting the DG. cFos-dependent expression of ChR2-mCherry occurs only in absence of dox. Representative mCherry expression (scale bar, 100 μm). (**L**) Mice were taken off-dox presocialization to allow engram labeling and put on dox afterward. All mice were sleep deprived, with one group receiving optogenetic activation before SM. (**M** and **N**) Socialization: Both groups preferred the stimulus. (**O** and **P**) SM: Mice with optogenetic activation showed no preference, indicating successful retrieval. (**Q** and **R**) cFos-tTA mice were injected with AAV9-TRE-ChR2-mCherry or AAV9-TRE-mCherry. Mice were sleep-deprived postsocialization and received laser stimulation before SM. (**S** and **T**) Socialization: Both groups preferentially explored the stimulus. (**U** and **V**) SM: ChR2^+^ mice showcased no preference, whereas ChR2^−^ mice preferred the stimulus. **/##*P* < 0.01, ***/###*P* < 0.001, ***/####*P* < 0.0001: paired *t* tests (C and D), one-sample *t* tests (E, H, J, N, P, T, and V), two-way repeated measures analysis of variance (RM-ANOVA) with Bonferroni (G, I, M, O, S, and U), unpaired *t* tests (J, P, and V). Data: means ± SEM. *n* = 9 to 12 per group. Detailed statistics: table S1. Created in BioRender. A. Sarma (2026) https://BioRender.com/6qq2jch and Adobe Illustrator.

To investigate the impact of SD on long-term SRM, we developed a protocol based on previous findings that adult male mice exhibit a strong initial preference for a novel male stimulus over a familiar male cagemate (Fig. 1A) (*8*, *9*). Successful SRM formation and retrieval would be demonstrated by a reduced preference for the stimulus mouse upon reexposure as familiarity with the previously encountered mice would result in equal exploration of both mice (Fig. 1B). Our results show that, during the socialization session, test mice explored the stimulus mice significantly more than the cagemate (Fig. 1C). However, when reexposed to the same mice during the social memory test next day, the test mouse explored both mice similarly (Fig. 1D). The strong preference for the stimulus mouse during the socialization session (Fig. 1E, left) and the lack of preference for the stimulus mouse over the cagemate during reexposure suggested successful SRM formation as the test mice no longer perceived the stimulus as novel (Fig. 1E, right). These observations indicate that the protocol allows us to successfully study SRM.

To determine whether SD affects SRM, we subjected the test mice to 6 hours of induced wakefulness immediately following socialization (Fig. 1F). As expected, during the socialization session, all test mice explored the stimulus mice significantly more than the cagemate, showing a preference for the novel social stimulus (Fig. 1, G and H). However, during the memory test 24 hours later, only the non–sleep-deprived mice explored the stimulus mouse and the cagemate similarly. In contrast, sleep-deprived mice still explored the stimulus mice significantly more than the cagemate (Fig. 1I). This implies that the sleep-deprived mice, unlike their non–sleep-deprived counterparts, had not lost their preference for the stimulus mice following repeated exposure, suggesting a weaker recognition memory (Fig. 1J). Overall, our results indicate that long-term social memories are vulnerable to SD. These findings align with the previously shown effect of SD on other long-term memories (*10*–*13*) and show that SD immediately posttraining (in our case, postsocialization) impairs the performance in a memory test later on.

### Optogenetic reactivation of DG engram cells restores SRM impaired by SD

Recent work by multiple laboratories has indicated that memory deficits associated with amnesia are often a result of suboptimal storage affecting information access and retrievability rather than a failure in memory storage (*14*–*18*). Therefore, we tested the hypothesis that SD following socialization might not lead to a loss of information but merely hampers the retrieval of memory in mice exposed to the SRM task. Previous research has shown that the hippocampus is highly sensitive to sleep and sleep loss (*19*–*24*) and that social recognition memories are tightly linked to hippocampal function (*25*–*28*). Moreover, the dentate gyrus (DG), a hippocampal subregion important for social memories is particularly vulnerable to the effects of SD on memories (*16*, *29*, *30*). Therefore, we specifically labeled engram cells in the DG when animals were taken off doxycycline (dox)–containing food to open the window for tagging (*31*). The animals were taken off dox 24 hours before the socialization session and kept off dox until immediately after socialization, corresponding with the start of the 6-hour SD period (Fig. 1, K and L). Labeling did not occur when the animals were kept on dox (Fig. 1, K and L). During the socialization session, both groups of sleep-deprived mice explored the stimulus mouse significantly more than the cagemate showing a significant preference (Fig. 1, M and N).

To investigate whether memory retrieval could be restored, we used optogenetic reactivation of the tagged memory engram in the DG. Five to 10 min before the memory test, the memory engram was optogenetically reactivated in one group of mice, while the other group was tethered to the cable without stimulation. During the memory test, only the sleep-deprived mice without optogenetic stimulation continued to show a strong preference for the stimulus mouse, exploring it more than the cagemate. This strong preference suggests that the test mouse failed to recognize the stimulus mouse, indicating impaired memory retrieval (Fig. 1, O and P). In contrast, the sleep-deprived mice that underwent optogenetic reactivation of DG engram cells explored both the stimulus mouse and the cagemate equally, showing successful retrieval of the previously formed social memory (Fig. 1, O and P). The tagged memory engram in the DG formed during socialization likely includes neurons activated during exploration of both the familiar cagemate and the novel stimulus mouse, reflecting the combined social context of that experience. The memory retrieved by optogenetic engram activation therefore corresponds to the familiarization of the previously novel mouse within the context that already includes the familiar cagemate. In this sense, the tagged ensemble represents the social experience as a whole rather than encoding the identity of only one mouse, while the behavioral outcome of its reactivation demonstrates that it contains the information necessary to recognize the previously novel individual. Thus, the key conclusion from our optogenetic experiments is not selectivity per se, but sufficiency, i.e., reactivation of this ensemble alone is enough to restore appropriate social discrimination after SD.

To confirm that the observed rescue of SRM depended on functional optogenetic reactivation of the tagged engram, we performed additional control experiments comparing mice with and without functional channelrhodopsin expression in the DG (Fig. 1, Q and R). During the socialization session, both groups explored the stimulus mouse significantly more than the familiar cagemate (Fig. 1, S and T). Following socialization, all mice were subjected to 6 hours of SD. Twenty-four hours later, both groups received optogenetic stimulation before the social memory test. During testing, only mice expressing functional channelrhodopsin explored the stimulus mouse and the familiar cagemate equally, indicating successful memory retrieval. In contrast, mice lacking functional channelrhodopsin continued to show a significant preference for the stimulus mouse (Fig. 1, U and V), similar to the retrieval deficits observed following SD in earlier experiments. These findings demonstrate that restoration of SRM requires functional optogenetic activation of the tagged DG engram. Together, this evidence suggests that SD-induced amnesia in social memories stems primarily from impaired retrieval. Although SD impedes access to SRM, the memory itself is not lost. Instead, it likely persists in a weakened, inaccessible state. Direct stimulation of the engram may strengthen these weakened connections, thereby restoring memory retrieval.

### Treatment with an FDA-approved PDE4 inhibitor restores social recognition memories impaired after SD

**Fig. 2.**
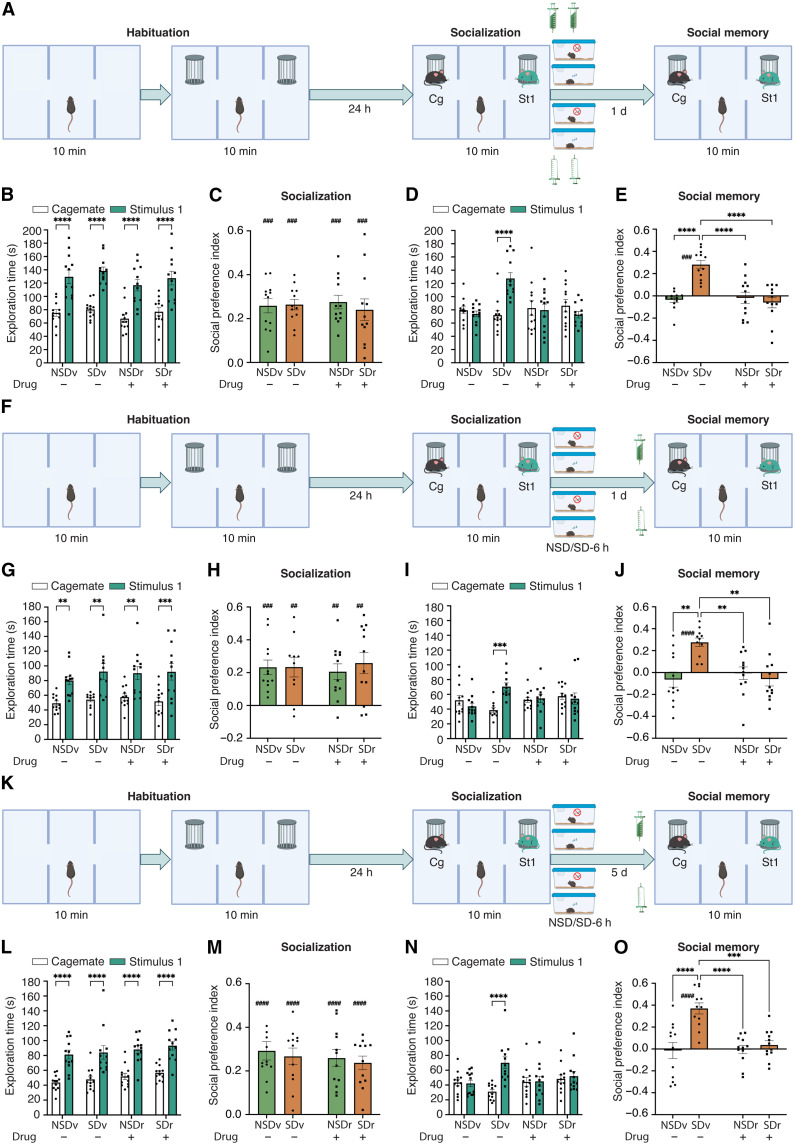
Treatment with roflumilast preceding the social memory test rescues the memory deficits caused by SD following socialization. (**A**) Test mice were habituated to an empty arena, followed by exposure to two empty wire cups. After 24 hours, the mice were exposed to a cagemate and a novel stimulus, followed by 6 hours of SD or an undisturbed rest period (NSD). Mice received intraperitoneal injections of roflumilast (0.03 mg/kg) or vehicle immediately after socialization and again 3 hours later. Social memory was assessed 24 hours after socialization. (d, days). (**B** and **C**) During socialization, all groups explored the stimulus more than the cagemate and showed a positive SPI. (**D** and **E**) During social memory, sleep-deprived mice treated with vehicle explored the stimulus more than the cagemate. In contrast, sleep-deprived mice treated with roflumilast explored both mice equally, indicating restored memory retrieval. (**F**) Similar to (A), except roflumilast or vehicle was administered 30 min before social memory. (**G** and **H**) During socialization, all groups explored the stimulus more than the cagemate. (**I** and **J**) During social memory, sleep-deprived mice treated with vehicle explored the stimulus significantly more, whereas sleep-deprived mice treated with roflumilast explored both mice equally indicating successful retrieval. (**K**) Similar to (F), but the social memory test occurred 5 days after socialization. (**L** and **M**) During socialization, all groups explored the stimulus more than the cagemate. (**N** and **O**) During social memory, sleep-deprived mice treated with vehicle preferred the stimulus, whereas sleep-deprived mice treated with roflumilast explored both mice equally, demonstrating restored memory retrieval. **/##*P* < 0.01, ***/###*P* < 0.001, and ****/####*P* < 0.0001 were calculated using one-sample *t* tests (C, E, H, J, M, and O), or two-way RM ANOVA with Bonferroni post hoc (B, D, G, I, J, L, N, and O). Data presented as means ± SEM. *n* = 10 to 12 per group. Detailed statistics: table S2. Created in BioRender. A. Sarma (2026) https://BioRender.com/2hlm5p9 and Adobe Illustrator.

To enhance the translational potential of our findings, we explored a noninvasive approach to address memory retrieval deficits. Previous studies indicate that elevated cyclic adenosine monophosphate (cAMP) levels are essential for hippocampus-dependent memory consolidation and retrieval, and impairments are often linked to reduced cAMP or increased activity of phosphodiesterase type 4 (PDE4), an enzyme that degrades cAMP (*11*, *32*–*36*). On the basis of these findings, we hypothesized that SD attenuates SRM consolidation and retrieval by decreasing cAMP levels through an increase in PDE4 activity. Therefore, we first tested whether PDE4 inhibition could prevent impairments in SRM consolidation induced by postsocialization SD. Immediately following the socialization session, the mice were either subjected to 6 hours of SD or left undisturbed in their home cages. Mice received intraperitoneal injections of either the clinically approved PDE4 inhibitor roflumilast or vehicle immediately after socialization and again 3 hours later (Fig. 2A). This timing was chosen to target the consolidation window following social experience and was based on previous studies (*11*, *24*, *37*). SRM was assessed during a social memory test performed the following day. During socialization, all mice showed a strong preference for the stimulus mouse over the cagemate (Fig. 2, B and C). During the social memory test, non–sleep-deprived mice displayed intact memory irrespective of treatment. In contrast, sleep-deprived mice that received vehicle treatment showed impaired memory retrieval, as indicated by increased exploration of the stimulus mouse. Sleep-deprived mice treated with roflumilast after socialization explored the stimulus mouse and the cagemate equally, indicating preserved SRM despite subsequent SD (Fig. 2, D and E). These findings are consistent with previous work on other memory types showing that hippocampus-dependent memory impairments following SD, at least for spatial memories, are associated with reduced cAMP signaling and increased PDE4 activity (*11*). Our results extend this framework by demonstrating that pharmacological PDE4 inhibition with roflumilast rescues SRM deficits when administered during the consolidation window.

Next, we tested whether inhibiting PDE4 with the roflumilast before the social memory test could restore SRM retrieval. As before, the mice were subjected to 6 hours of SD after the socialization session. Then, 30 min before the social memory test next day, they were injected intraperitoneally with roflumilast or vehicle solution (Fig. 2F). During the socialization session, all mice exhibited a strong preference for the stimulus mouse, spending considerably more time interacting with it compared to the cagemate (Fig. 2, G and H). During the social memory test, the control mice showed no preference for the stimulus mouse, indicating successful retrieval of the memory following the socialization session. Moreover, the drug treatment did not affect memory performance in the non–sleep-deprived mice (Fig. 2, I and J). However, sleep-deprived mice that received vehicle treatment continued to explore the stimulus mouse more than the cagemate, showing impaired memory retrieval (Fig. 2, I and J). In contrast, sleep-deprived mice that were treated with roflumilast explored both the stimulus mouse and the cagemate equally, indicating successful memory retrieval (Fig. 2, I and J).

In amnesia mouse models including those for early stages of Alzheimer’s disease, memories persist several days after the initial training in an inaccessible state that can only be retrieved with targeted interventions (*14*, *15*, *17*). Therefore, we hypothesized that a memory formed after socialization also persists several days after the initial encounter. However, SD may render this memory difficult to retrieve. To investigate this hypothesis, we tested memory retrieval 5 days postsocialization in a new batch of mice (Fig. 2K). During the socialization session, all the mice spent more time exploring the stimulus mice in comparison to the cagemate (Fig. 2, L and M). During the social memory test, the non–sleep-deprived mice that received vehicle or roflumilast treatment explored the stimulus and cagemate equally, showcasing successful memory retrieval. As expected, sleep-deprived mice that received vehicle treatment showed a clear preference for the stimulus mouse, indicating impaired memory retrieval (Fig. 2, N and O). In contrast, sleep-deprived mice treated with roflumilast before the memory test explored the stimulus mouse and cagemate equally, suggesting successful memory retrieval (Fig. 2, N and O). These findings showcase two key outcomes: First, non–sleep-deprived mice retain the memory of encountering the stimulus mice even 5 days postsocialization. Second, these memories persist in sleep-deprived mice in a suboptimally stored condition and can be retrieved by systemic administration of roflumilast either during the consolidation window immediately following socialization or directly preceding the social memory test.

### Optogenetic stimulation of DG engram cells enables persistent retrieval of social memory several days later by increasing engram reactivation

**Fig. 3.**
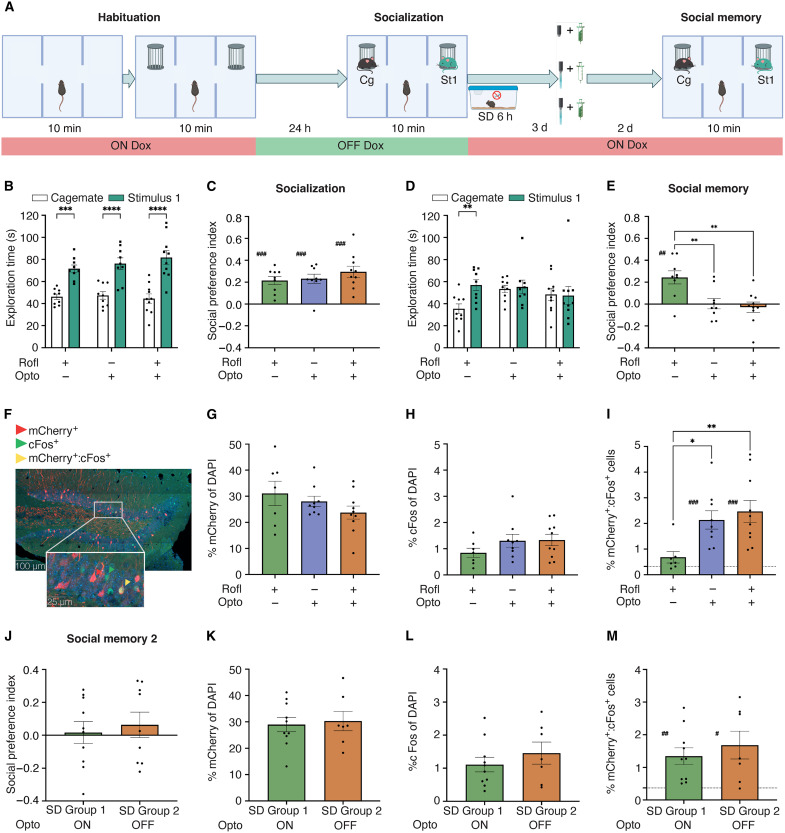
Social memories formed under SD conditions can be made persistently accessible by optogenetics alone and in combination with roflumilast treatment. (**A**) Twenty-four hours posthabituation, cFos-tTa mice were exposed to a cagemate and a novel stimulus followed by 6 hours of SD. Mice were taken off dox 24 hours before socialization for engram labeling and returned to dox immediately after. Three days after socialization, mice received optogenetic activation (i), roflumilast (ii), or a combination of both (iii). Social memory was assessed 5 days postsocialization. (**B** and **C**) During socialization, all groups explored the stimulus more than the cagemate. (**D** and **E**) During social memory, groups (i) and (iii) explored both mice equally, indicating successful retrieval. (**F**) Representative DG images showing DAPI (blue), mCherry (red), cFos (green), and mCherry^+^ cFos^+^ (yellow). Scale bar, 100 μm; zoomed-in view, 25 μm. (**G** and **H**) No group differences were observed in %mCherry or cFos in the DG. (**I**) mCherry^+^ cFos^+^ colocalization was lower in (ii) compared to (i) and (iii). (**J**) Following successful retrieval in (A), mice from Fig. 1 (L to P) were retested 6 days after socialization using a crossover design. Groups with and without prior optogenetic stimulation were switched. During social memory, mice showed no preference indicating successful retrieval. (**K** and **L**) No group differences were observed in mCherry or cFos in the DG. (**M**) Laser stimulation delivered 1 or 6 days after socialization resulted in mCherry^+^ cFos^+^ colocalization above chance. */#*P* < 0.05, **/##*P* < 0.01, ***/###*P* < 0.001, ****P < 0.0001 were calculated using one-sample *t* tests (C, E, I, J, and M), two-way RM ANOVA with Bonferroni post hoc (B and D), one-way ANOVA (G, H, and E), Kruskal-Wallis with Dunn’s post hoc (I), Dunnett’s post hoc (E), unpaired *t* tests (J), and two-tailed *t* tests (K and L). Data are presented as means ± SEM. *n* = 9 to 10 (B to E and J); *n* = 7 to 10 (G to I and K to M). Detailed statistics: table S3. Created in BioRender. A. Sarma (2026) https://BioRender.com/88f35zh and Adobe Illustrator.

Previous studies have shown that optogenetic stimulation can temporarily restore access to fear and object memories formed under suboptimal conditions. However, these effects have often been short-lived, lacking long-lasting restoration (*14*, *15*, *38*). So far, we observed successful memory retrieval in previously sleep-deprived mice only when roflumilast was administered either immediately after socialization or before the memory test, or when optogenetic stimulation was delivered shortly before memory testing. We then aimed to persistently restore long-term access to SRM, allowing for successful natural retrieval several days later. To this end, we combined optogenetic stimulation of the engram formed under SD conditions with roflumilast administration, a combination that restored persistent access to spatial memories (*16*). Specifically, we exposed a new batch of mice to a socialization session, tagging DG neurons activated during learning, followed by 6 hours of SD. Three days later, the animals were subjected to one of three experimental conditions. In the first group, the mice were connected to optogenetic cables but did not receive laser stimulation, and they were given an intraperitoneal injection of roflumilast 3 hours after this mock stimulation. The second group received optogenetic activation of the tagged engram cells, followed by an intraperitoneal injection of vehicle 3 hours after the optogenetic activation. The third group received optogenetic activation followed by an intraperitoneal injection of roflumilast 3 hours later (Fig. 3A). The 3-hour time point was chosen on the basis of prior work showing that PDE4 inhibition boosts memory consolidation when administered 3 to 5.5 hours after learning (*16*, *39*–*41*).

This, in combination with our observation that roflumilast administered after socialization rescues memory deficits in Fig. 2 (A to E), supports the idea that optogenetic reactivation, in this combinatorial approach, may serve as a learning-like event that can be stabilized through cAMP-dependent mechanisms. Two days later, the memory test was conducted without any further interventions before the test. During socialization, all test mice exhibited a strong preference for the stimulus mouse, exploring it significantly more than the cagemate (Fig. 3, B and C). However, during the memory test, the mice that received roflumilast treatment only explored the stimulus mouse more (Fig. 3D). This preference toward the stimulus mouse over the cagemate indicated impaired memory retrieval, suggesting that roflumilast alone was not sufficient for reconsolidation and persistent access to SRM (Fig. 3E). This was consistent with our expectations as the elevation of cAMP levels cannot aid in memory reconsolidation if no memory-related processes have been induced (*16*). Unexpectedly, mice receiving optogenetic stimulation only showed no preference toward the stimulus mouse during the memory test, suggesting successful retrieval (Fig. 3, D and E). Likewise, the group receiving both optogenetic activation and roflumilast also explored both mice similarly, indicating successful retrieval (Fig. 3, D and E). Together, these findings imply that the optogenetic activation alone may be sufficient to restore long-term SRM retrieval, as indicated by the similar exploration times observed in both the optogenetics-only and combination groups. While at the first glance, it seems that the optogenetic stimulation and the combinatorial approach leads to similar memory performance, it may very well be that the benefit of combining engram activation and drug treatment would become apparent if tested at a later time point.

Successful memory retrieval in amnesia models has previously shown to be associated with enhanced engram reactivation (*14*, *15*, *17*, *18*). Having observed successful memory retrieval with optogenetics only and in combination with pharmacological treatment in our study, we sought to investigate whether these manipulations also affected engram reactivation in the DG. To do this, we counted mCherry- and cFos-positive cells (Fig. 3F). The mCherry-positive cells represent neurons that were tagged during the socialization session, marking them part of the original engram associated with the social memory. The cFos-positive cells, on the other hand, reflect the neurons activated during the social memory test. A higher degree of colocalization between the two is expected upon successful memory retrieval (*14*, *15*, *17*, *42*, *43*).

Therefore, we expected a higher degree of colocalization between these two markers in the optogenetics only and the combinatorial group in comparison to the sleep-deprived mice that received drug treatment only. Our analysis revealed that the percentages of tagged mCherry (Fig. 3G and fig. S2, A and D) and cFos-positive cells were similar across all experimental groups (Fig. 3H and fig. S2, B and E). However, the optogenetic-only and combination treatment groups exhibited significantly higher colocalization of mCherry and cFos in the DG compared to the roflumilast-only group, which did not show colocalization above chance levels (Fig. 3I). This increase in colocalization was predominantly driven by increased engram reactivation in the superior blade of the DG (fig. S2, C and F). The aforementioned results suggests that SD after the socialization phase results in attenuated engram reactivation during testing and that optogenetic activation or in combination with roflumilast can restore this reactivation.

On the basis of these results, we determined whether the restoration by optogenetic stimulation alone could persist over even longer intervals (i.e., 6 days postsocialization). To do this, we retested the mice from Fig. 1 (L to P) using a crossover design. In this experiment, group 1, which had not previously received optogenetic stimulation, now received it. Conversely, group 2, which had received stimulation previously, did not undergo further stimulation. Both groups were tested 6 days after the socialization session (Fig. 3, J to M). Both groups explored the stimulus and cagemate mice equally during the memory test. These observations indicated successful retrieval of social memory in both cases (Fig. 3J). This suggests that a single optogenetic activation is sufficient to restore access to the memory engram even several days later in both cases despite the different points at which the engram was optogenetically reactivated. The findings were further supported by colocalization studies: We found that both groups exhibited similar colocalization levels between mCherry (Fig. 3K and fig. S2, G and J) and cFos (Fig. 3L and fig. S2, H and K), with colocalization significantly higher than chance in both the superior and inferior DG (Fig. 3M and fig. S2, I and L). Together, the findings show that long-term social memories made inaccessible by SD can be persistently restored by optogenetic activation, which is paralleled by increased engram reactivation.

While it remains unclear why social memories formed under suboptimal conditions can be persistently restored, our findings suggest that they may differ from other long-term memories. Unlike previous studies reporting transient restoration of fear and object memories, we observed that a single optogenetic activation of DG engram cells enables persistent retrieval of SRM several days later. This distinction might reflect unique features of social memories, such as differences in the underlying signaling pathways or their distinct sensitivity to the effects of SD.

### Optogenetic reactivation of DG engram cells restores access to memories encoding individual social experiences

**Fig. 4.**
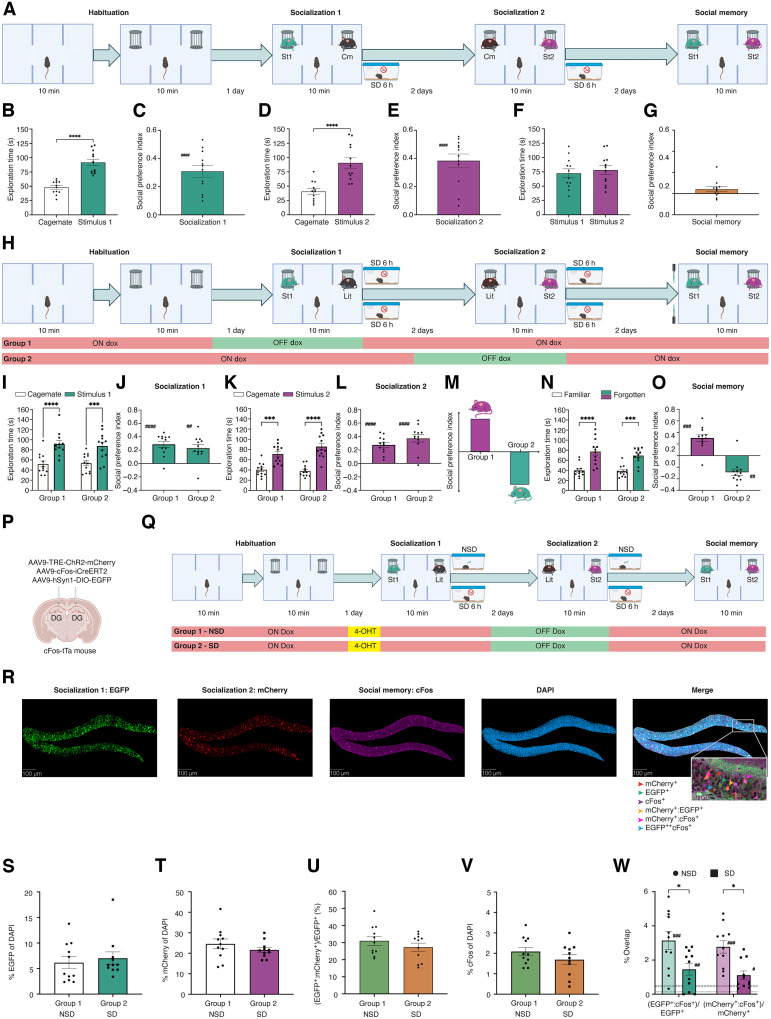
Optogenetic engram reactivation allows previously sleep-deprived mice to discriminate between individual social experiences. (**A**) Mice were habituated to the arena with two wire cups. After 24 hours, the mice were exposed to the cagemate and stimulus-1 (Soc1), followed by 6 hours of SD. Forty-eight hours later, the mice underwent a second socialization with stimulus-2 (Soc2), again followed by 6 hours of SD. SM was tested 6 days after Soc1 with both stimuli presented in their original locations. (**B** and **C**) Soc1: The mice preferentially explored stimulus-1. (**D** and **E**) Soc2: The mice preferentially explored stimulus-2. (**F** and **G**) SM: The mice showed no preference. (**H**) Similar to (A) with Soc1 tagged in group 1 and Soc2 tagged in group 2. Both groups received optogenetic activation before SM. (**I** and **J**) Soc1: Both groups preferentially explored stimulus-1. (**K** and **L**) Soc2: Both groups preferentially explored stimulus-2. (**M**) Expectation: In group 1, optogenetic stimulation allows mice to retrieve the memory of stimulus-1 and therefore preferentially explore stimulus-2 during the SM. In group 2, the opposite pattern is expected. (**N** and **O**) SM: Both groups preferentially explore the untagged stimulus. (**P**) Soc1 was tagged using a tamoxifen-inducible Cre-dependent EGFP system. Soc2 was tagged using a dox-regulated cFos-tTA/TRE-mCherry system. (**Q**) Tamoxifen was administered 4 hours before Soc1 allowing EGFP expression, and dox was removed 24 hours before Soc2 allowing mCherry expression in sleep-deprived and non–sleep-deprived mice. (**R**) Representative DG images showing EGFP, mCherry, cFos, DAPI, and merged channels (Scale bar, 100 μm); zoomed-in inset (Scale bar, 20 μm) (**S** to **V**) %EGFP^+^, mCherry^+^, EGFP^+^mCherry^+^, and cFos^+^ cells did not differ between non–sleep-deprived and sleep-deprived mice. (**W**) SM: Both non–sleep-deprived and sleep-deprived groups were above chance for EGFP^+^cFos^+^ (dotted line) and mCherry^+^cFos^+^ colocalization (dashed line); however, sleep-deprived mice showed significantly lower reactivation than non–sleep-deprived mice. #/*P < 0.05, ##*P* < 0.01, ###/***P < 0.001, ****P < 0.0001: paired *t* tests (B, D, and F); one-sample *t* tests (C, E, G, J, and L); two-way RM-ANOVA with Bonferroni (I, K, N, and W); Wilcoxon signed-rank tests (O and W); unpaired *t* tests (T to V); Mann-Whitney *U* tests (S). Data: means ± SEM; *n* = 12 per group. Detailed statistics: table S4. Created in BioRender. A. Sarma (2026) https://BioRender.com/31f3d0u and Adobe Illustrator.

Our results suggest that a single social memory formed under suboptimal conditions can be persistently retrieved several days later. However, more often than not, we encounter multiple social experiences over time within the same environment. The ability to recall and distinguish each experience even when experienced within the same context is essential for navigating social relationships (*44*–*46*). This prompted us to investigate whether mice have the ability to distinguish multiple social experiences formed in similar contexts and, if so, whether SD affects this process. We therefore extended the SRM protocol to include two socialization sessions, each with a novel stimulus mouse. During the first socialization session, each test mouse was introduced to a novel stimulus mouse, with its familiar cagemate placed on the opposite side of a three-chambered arena. In the second socialization session, the position of the cagemate and a novel stimulus mouse was switched. During the social memory test, 2 days later, the test mice were exposed to both stimulus mice. The stimulus mice were placed in the same positions they had occupied during their respective socialization sessions. One group of test mice was sleep deprived after both socialization sessions, while the other group was allowed to sleep (Fig. 4A and fig. S3A). During the first and second socialization sessions, all test mice exhibited a strong preference for the stimulus mice, exploring them significantly more than the cagemate (Fig. 4, B to E, and fig. S3, B to E). During the memory test, the sleep-deprived as well as non–sleep-deprived mice explored both stimulus mice equally (Fig. 4, F and G, and fig. S3, F and G). However, the sleep-deprived mice spent significantly more time exploring the stimulus mice toward levels seen during the original socialization sessions (fig. S3, H and I). This observation is in line with data shown in for example, Figs. 1I and 2I. On the basis of these results, we concluded that sleep-deprived mice, unlike their non–sleep-deprived counterparts, were unable to recall either social experience, evidenced by increased exploration of both the stimulus mice. This finding raises the intriguing question of whether information about both individual experiences are present in the brain. At the same time, the data do not address whether the mice can distinguish between the two social experiences. To address both questions, we decided to replicate the study using our engram technology to selectively tag either of the two socialization sessions. Specifically, we trained two groups of mice in which we tagged either the first or second socialization session (Fig. 4H). The two groups of test mice were sleep deprived after both socialization sessions. During the first and second socialization sessions, all test mice showed a clear preference for the novel stimulus mouse, exploring it significantly more than the familiar cagemate (Fig. 4, I and J, for the first socialization and Fig. 4, K and L, for the second socialization). During the social memory test, the mice were exposed to both stimulus mice. We predicted three possible outcomes for this experiment. First, if no engram was present for the tagged memory, optogenetic activation would have no effect. In this case, the test mice would show increased exploration times for both stimulus mice, indicating a failure to recall the memory. Second, if the engram stored information about both social experiences, optogenetic activation would reduce exploration times for both stimulus mice as both would be perceived as familiar. However, if the engram contained information specific to a single social experience, optogenetic activation would selectively retrieve the tagged memory resulting in the test mice exploring the untagged stimulus mouse more than the tagged one. This would indicate that optogenetic engram reactivation successfully and specifically recalled the memory of the tagged stimulus mouse (Fig. 4M). In the social memory test, both groups explored the untagged stimulus mouse significantly more than the tagged one, aligning with our third predicted outcome (Fig. 4N). This preference suggests that reactivation enabled the test mice to recall the tagged memory, while the untagged stimulus mouse was not recognized, indicating a failure to retrieve the memory associated with it (Fig. 4O). Together, our results demonstrate that mice can distinguish between multiple social experiences formed in similar contexts. The use of engram technologies to selectively recall the tagged memory indicates that information about multiple social experiences is preserved in the brain, even when formed under SD conditions.

### SD attenuates reactivation of DG engrams encoding multiple social experiences

Our behavioral experiments shown in Fig. 4 (A to O) demonstrate that mice can distinguish between multiple social experiences and that SD primarily disrupts access to these memories. These findings raise the question how individual social experiences are represented at the level of the DG. In particular, it remains unclear how SD affects the subsequent reactivation of neuronal ensembles associated with subsequent but unique social encounters.

To directly examine how individual social experiences are encoded and subsequently reactivated, we combined our existing activity-dependent tagging strategy with an intersectional viral approach, allowing us to independently label neuronal populations activated during two distinct social experiences within the same animal. Specifically, we combined the cFos-tetracycline-transcriptional-transactivator (tTA)/tetracycline response element (TRE) system, which is regulated by dox, with a tamoxifen-inducible Cre-based system, thereby enabling temporally separated labeling of two experiences using different fluorescent markers. Mice were bilaterally injected in the DG with a viral cocktail consisting of a TRE-driven mCherry construct and a Cre-dependent enhanced green fluorescent protein (EGFP) reporter, together with a cFos-driven CreERT2 construct (Fig. 4P). In this configuration, neuronal activity occurring in the absence of dox resulted in tTA-mediated expression of mCherry, whereas neuronal activity occurring in the presence of tamoxifen resulted in Cre-mediated recombination and expression of EGFP. This dual-tagging strategy allowed us to label neurons activated during two separate socialization sessions using distinct fluorophores within the same DG. We then applied the same two-session socialization paradigm described in Fig. 4H. Briefly, mice underwent two socialization sessions with different stimulus mice presented in opposing chambers of the three-chamber arena. In this experiment, however, the two social experiences were differentially tagged: Neurons activated during the first socialization session were labeled using the Cre-dependent EGFP system, whereas neurons activated during the second socialization session were labeled using the tTA-dependent mCherry system (Fig. 4Q). One group of mice was allowed to sleep normally after each socialization session (i.e., non–sleep-deprived group), while a second group was subjected to 6 hours of SD immediately following each socialization session (i.e., sleep-deprived group).

Behaviorally, both non–sleep-deprived and sleep-deprived mice showed outcomes similar to those observed in the previous study (i.e., fig. S3, H and I). During the social memory test, mice from both groups explored stimulus mouse 1 and stimulus mouse 2 equally, indicating an absence of preferential exploration of either stimulus (fig. S3, O and P). Consistent with our earlier findings, sleep-deprived mice exhibited significantly higher total exploration times toward both stimulus mice compared to non–sleep-deprived controls during the test session (fig. S3P). This increase in overall exploration suggests impaired retrieval of both social memories following SD despite the presence of information related to both experiences.

At the cellular level, we first quantified the proportion of neurons labeled with EGFP or mCherry in the DG. The percentage of EGFP-positive cells, representing neurons activated during the first socialization session, did not differ between non–sleep-deprived and sleep-deprived mice (Fig. 4S). Similarly, the percentage of mCherry-positive cells, corresponding to neurons activated during the second socialization session, was comparable between the non–sleep-deprived and sleep-deprived groups (Fig. 4T). These observations indicate that SD does not alter the overall recruitment of DG neurons during either social experience regardless of the tagging system used.

Next, we assessed the degree of overlap between the neuronal ensembles associated with the two social experiences by quantifying the proportion of neurons coexpressing EGFP and mCherry. The extent of overlap between EGFP- and mCherry-positive populations was around 30% and comparable between non–sleep-deprived and sleep-deprived mice, with no significant differences observed between groups (Fig. 4U). Despite this partial overlap, our optogenetic experiments described above in Fig. 4 (N and O) demonstrate that reactivation of a tagged ensemble selectively retrieves the individual social experience, in support of the pattern separation function of the DG. Furthermore, this suggests that SD does not alter the degree of overlap between the neuronal ensembles associated with the two social experiences. We then examined neuronal activation during the social memory test by quantifying cFos expression in the DG. The overall proportion of cFos-positive neurons relative to total 4′,6-diamidino-2-phenylindole (DAPI)–labeled cells did not differ between non–sleep-deprived and sleep-deprived mice (Fig. 4V), indicating that a brief period of SD following training does not globally suppress DG activity in terms of cFos expression during subsequent memory retrieval.

Last, to determine whether SD affects experience-specific engram reactivation, we quantified the overlap between cFos expression and each of the tagged engram populations. Chance overlap was calculated as the product of the single-label fractions relative to DAPI, pooled across all animals as neither engram labeling nor overall DG activity differed between groups. In non–sleep-deprived mice, cFos expression during the memory test significantly overlapped with both EGFP- and mCherry-labeled populations above chance levels, indicating reactivation of neuronal ensembles associated with both social experiences. In contrast, sleep-deprived mice showed significantly reduced overlap between cFos and each tagged population, although colocalization remained above chance in both groups (Fig. 4W). This reduction was observed for both the EGFP-labeled ensemble corresponding to the first social experience and the mCherry-labeled ensemble corresponding to the second experience.

Together, using multiple cFos-driven engram tagging approaches in a single animal, these findings indicate that SD does not alter the formation of neuronal ensembles encoding multiple social experiences nor does it change the magnitude of overlap of DG memory engrams associated with consecutive social experiences in the same context. Instead, SD selectively attenuates the reactivation of these experience-specific engrams during memory retrieval. This provides a cellular explanation for the behavioral phenotype we observed in mice that were previously sleep deprived, wherein information about multiple social experiences is preserved but remains inaccessible under natural retrieval conditions.

## DISCUSSION

In this study, we show that mice can form persistent social memories and recognize previously encountered individuals several days later and that multiple social experiences formed in the same context are retained as distinct memories. SD during the consolidation window disrupted long-term SRM. However, this consolidation impairment did not reflect loss of the memory itself but rather a failure to access it at the time of retrieval. At the mechanistic level, this interpretation is reinforced by our observation that social memory performance could be restored transiently by pharmacological inhibition of PDE4 signaling using the Food and Drug Administration (FDA)–approved PDE4 inhibitor roflumilast preceding the test session and more persistently by optogenetic reactivation of DG engram cells. Furthermore, roflumilast treatment during SD made memory consolidation resilient to the negative impact of SD. Moreover, by independently tagging neuronal ensembles associated with consecutive social experiences, we show that SD does not impair or alter engram formation or change the degree of overlap between ensembles, but instead selectively attenuates experience-specific engram reactivation during retrieval. Together, these findings highlight a critical role for the hippocampal DG in the formation of long-term social memory and discrimination between individual social experiences when they take place in a single context. Moreover, our findings extend previous work on spatial memories (*16*) highlighting that SD does not lead to information loss but rather leads to a failure to successfully recall the information stored under SD conditions at a time and place where this would be essential.

This behavioral phenotype cannot be explained by nonspecific effects of SD on locomotion, anxiety, or stress. SD was applied after socialization and not immediately before memory testing, making acute effects on exploration during the test unlikely. Consistent with this, total social exploration during the memory test was not reduced in sleep-deprived mice across experiments. Instead, SD selectively altered how exploration was distributed between the stimulus mouse and the familiar cagemate, with impaired retrieval consistently associated with increased exploration of the stimulus mouse. This pattern argues against a generalized motivational or anxiety-related deficit and instead points to a memory-specific impairment in retrieval. In line with this, previous work has shown that short-term SD using gentle handling does not elevate glucocorticoid levels beyond normal circadian fluctuations (*47*) and that loss of sleep leads to amnesia even when glucocorticoid synthesis is blocked during SD (*48*). Moreover, extensive work by the laboratories of Roozendaal and McGaugh has shown that moderate acute glucocorticoid signaling around the time of learning would facilitate it rather than impair memory consolidation (*49*). Together, these findings support the conclusion that SD rather than stress leads to amnesia.

Given the strong dependence of long-term SRM on sleep, an important next step is to understand which aspects of sleep support the stability of DG-dependent social representations. Sleep has recently been shown to support social memory through multiple, stage-specific mechanisms. Hippocampal reactivation during rapid eye movement (REM) sleep promotes the consolidation of SRM (*50*), whereas coordinated hippocampal activity during non-REM (NREM) sleep contributes to the stabilization of memory representations (*51*). These findings suggest that social memory consolidation is unlikely to rely on a single sleep component but instead emerges from complementary processes across distinct sleep stages. An important direction for future work will therefore be to determine how REM- and NREM-associated mechanisms contribute to the formation, strengthening, and later accessibility of social memories, and whether disruption of specific sleep components leads to separable behavioral outcomes. At the network level, these processes likely involve the reactivation of neuronal activity patterns following learning, which has been observed during sharp-wave ripple events in the hippocampus, where coordinated neuronal activity can reinstate patterns formed during prior experience. Reactivation of neuronal ensembles across postencoding periods during sleep has been suggested to support consolidation, with the same populations of cells being reactivated during later recall (*52*). Recent work shows that although sharp-wave ripples remain present during SD, the reactivation and replay of neuronal firing patterns during these events are diminished and, in some cases, nearly absent (*53*), indicating that the occurrence of ripples alone is not sufficient and that the coordinated reinstatement of activity patterns may be disrupted under SD conditions. Within this framework, our results are consistent with the idea that SD impairs retrieval by disrupting experience-specific engram reactivation rather than preventing engram formation. The reduced overlap between tagged ensembles and cFos activity at test days later supports this interpretation and aligns with the idea that postencoding reactivation might contribute to the stabilization and later recall of memory representations. Our rescue experiments such as optogenetic reactivation may bypass reduced endogenous reactivation by directly reinstating activity in the tagged DG ensemble, thereby restoring access to the stored memory. In parallel, PDE4 inhibition may enhance cAMP-dependent signaling pathways known to support synaptic plasticity and long-term potentiation, processes that are closely linked to memory consolidation and retrieval (*54*). Future studies combining activity-dependent tagging with recordings of hippocampal activity during sleep and SD will be required to directly test these relationships.

Optogenetic reactivation alone in our paradigm is sufficient to give long-term rescue of social memories even in the absence of a drug treatment (Fig. 3, E and J). A possible explanation for our findings could be that SD in our social recognition paradigm primarily shifts the memory into an impaired-access state that remains close to the retrieval threshold such that a single coordinated ensemble reactivation is sufficient to reestablish subsequent cue-driven access. The persistent restoration of access we see in social but not other memories [e.g., object-location memories (*16*)] could be because different kinds of memories rely on specific (sub)regions to a varying degree (*25*, *55*, *56*). This distinction may contribute to how severely different types of memories are affected by SD (*21*). Future studies should include loss-of-function experiments to investigate whether manipulating neuronal circuits within these brain (sub)regions during SD alters the effect of optogenetic engram reactivation in the previously sleep-deprived mice.

Together, these results support the growing notion that the DG is involved in pattern separation and plays a critical role in distinguishing similar events across time (*57*, *58*). Our data suggest that this function might extend to social memories, where multiple individual experiences must be retained and distinguished across time. While our findings demonstrate that optogenetic reactivation restores access to experience-specific engrams, they do not establish that individual neurons encode social identity in a strictly stimulus-specific manner. Rather, our data show that mice store information about multiple social experiences and that reactivation of a tagged DG ensemble can selectively restore access to one of these experiences in sleep-deprived mice. An important question that remains is how individual social identities are represented within these ensembles and how different hippocampal subregions such as the DG, area CA2, and CA1 interact to maintain and retrieve individual identities across multiple social experiences (*8*, *28*, *55*). Future studies could investigate this by using approaches that enable identification of stimulus-specific neuronal representations within the DG, such as the activity-dependent reactivation and calcium imaging approaches used by Okuyama *et al.* (*28*) to identify individual-specific neurons in ventral CA1. Toward this end, the application of multiple engram tagging systems in single individuals will play an essential role moving forward (*59*–*61*), including carefully deciding on the promoter used to mediate tagging as highlighted in unpublished work (*59*), and how the brain at the network level orchestrates successful recall of individual social experiences in place and time.

## MATERIALS AND METHODS

### Mice

Pharmacological and SD studies without light stimulation (Figs. 1, A to J, 2, and 4, A to G; and fig. S1) were done with C57Bl6/J male mice bred in our facility (first pairs obtained from Charles River) or with mice obtained from Charles River. For engram-tagging studies (Figs. 1, K to V, 3, and 4, H to W; and figs. S2 and S3), we used hemizygous cFos-tTA/cFos-shEGFP (enhanced green fluorescent protein-targeting short hairpin RNA) male mice on a C57BI6/J background. The mice were bred in our facility (first breeding pairs from the Jackson Laboratories). The transgenic mice express a tTA under the control of a cFos promoter, originally developed by M. Mayford. Wild-type littermates of the transgenic mice served as familiar animals, and male C57Bl6/J mice from Charles River were used as unfamiliar (novel) stimulus mice. In addition, for pilots 1 and 2, male A/J mice were obtained from Envigo (USA) at the age of 6 to 7 weeks and used as stimulus mice. The study was conducted on male mice because of the major impact of the estrous cycle on structural plasticity of neurons (*62*). cFos-tTA/cFos-shEGFP test mice (Figs. 1, K to V, 3, and 4, H to W; and figs. S2 and S3) were pair-housed with their littermates in compartmentalized cages, 42 cm by 26 cm by 14.5 cm (length by width by height). The mice could see and smell each other through a transparent plastic partition with a thin horizontal gap at the bottom. Any physical interaction that could harm the test mouse or the optic fiber implant was not possible. Wild-type C57Bl6/J test mice (Fig. 1, A to J, 2, and 4, A to G; and fig. S1) were pair-housed with their littermates in poly-carb clear cages with stainless steel wired lids. The stimulus C57BI6/J mice were housed in groups of two or three in a separate housing cluster in similar poly-carb clear cages. All the animals were provided with a paper roll, nesting material and sawdust as bedding and housed on a 12-hour light:12-hour dark cycle with ad libitum access to food and water. Initially, all mice were fed regular chow (Altromin). One week before surgery, cFos-tTA/cFos-shEGFP mice were switched to dox chow (40 mg/kg, Envigo) and then returned to regular chow 1 day before engram labeling. After labeling, their diet was changed to high-concentration dox chow (1000 mg/kg, Envigo) to prevent further labeling, maintained until the end of the experiment. All the test mice and the littermates were 2 to 5 months during testing, which took place at the beginning of the light phase. All procedures were approved by the national Central Authority for Scientific Procedures on Animals (CCD) and the Institutional Animal Welfare Body of University of Groningen (license numbers: AVD1050020209627 and AVD10500202518727) and conform Directive 2010/63/EU.

### Virus construct and packaging

To label memory engrams (Fig. 1, K to P, 3, and 4, H to O; and fig. S2), we bilaterally injected the adeno-associated virus (AVV9-TRE-ChR2-mCherry, titer: 3.75 × 10^14^, 200 nl; Penn Vector Core, Philadelphia, USA) in the DG of cFos-tTA/cFos-shEGFP mice. The virus was injected at the following coordinates: −2.0 mm anterior-posterior (AP), ±1.3 mm medial-lateral (ML), and −1.8 mm dorsal-ventral (DV). As previously described (*16*), channelrhodopsin-2 (ChR2) and mCherry expression was controlled by the TRE. This allowed the cFos expression to drive the expression of tTA, which in turn led to the expression of ChR2 and mCherry. Dox-containing chow suppressed the expression of ChR2 and mCherry. Therefore, cFos expression was only driving the transcription of ChR2 and mCherry in the absence of dox food.

For experiments examining the functional requirement of optogenetic engram reactivation (Fig. 1, Q to V), control mice received an alternative TRE-driven virus lacking channelrhodopsin. Specifically, the mice were bilaterally injected with AAV9-TRE-hM4D(Gi)-mCherry (200 nl per hemisphere; Viral Vector Facility, University of Zurich, Switzerland). These mice underwent identical surgical, behavioral, and optical stimulation procedures but did not express functional ChR2 in the DG. This approach ensured that any rescue of SRM depended specifically on optogenetic activation of ChR2-expressing engram cells rather than nonspecific effects of viral expression, optical stimulation, or experimental handling.

For experiments involving independent tagging of two consecutive social experiences within the same animal (Fig. 4, P to W, and fig. S3, J and K), a dual, activity-dependent viral labeling strategy was used. Mice were bilaterally injected into the DG with a viral cocktail consisting of a TRE-driven reporter, a Cre-dependent reporter, and a cFos-driven inducible recombinase. Specifically, the cocktail contained AAV9-TRE-mCherry (titer: 3.75 × 10^14^), AAV9–cFos-CreERT2 (titer: 2.43 × 10^10^, VVF), and AAV9-hsyn1-DIO-EGFP (titer: 8.5 × 10^12^, Addgene). All viruses were injected as a single cocktail to a total volume of 450 nl per hemisphere at the same stereotaxic coordinates as described above (−2.0 mm AP, ±1.3 mm ML, and −1.8 mm DV). In this configuration, neuronal activity occurring in the absence of dox resulted in tTA-mediated expression of mCherry via the TRE system, whereas neuronal activity occurring in the presence of tamoxifen resulted in CreERT2-dependent recombination and permanent EGFP expression.

### Viral injection and fiber optic implants surgery

All surgeries were conducted using a stereotactic apparatus (Kopf instruments) and followed a previously described protocol (*16*). Mice were anesthetized with isoflurane (1.5 to 2%) and remained on a heating pad throughout the surgery. At the start of the surgery, the mice were subcutaneously injected with carprofen (0.1 ml/10 g), and 0.1 ml of lidocaine was applied locally (subcutaneously) at the surgery site on the top of the skull. Small bilateral holes were drilled in the skull using a 0.5-mm diameter microdrill at the appropriate locations. A 10-μl Hamilton microsyringe was used to introduce the virus to the DG (see below for coordinates). The needle was first lowered to a pocket site where it remained for 1 min. It was then lifted to the injection site where it remained for 1 min before starting the injection. The flow rate was set to 70 nl/min and was controlled by a microinjection pump (Micro4; WPI). A total of 200 nl of the virus was injected per hemisphere. After the viral injection, the needle remained at the injection site for 2 min before it was removed from the brain. Optical fibers were mounted on the skull (see coordinates below) and stabilized with cement (C&B metabond) and two screws (0.96-cm diameter). At the end of the surgery, the mice were injected with 0.1 ml of saline (0.9%). All animals were allowed at least 1 week to recover before the start of the behavioral experiments. The coordinates for viral injection were as follows: −2.0 mm AP, ±1.3 mm ML, and −1.8 mm DV, and a pocket was made at −1.9 mm DV. The pocket is a small area underneath the injection site. It allows the virus to easily spread in the DG with the minimal pressure buildup. The optic fiber, if used, was mounted at −2.0 mm AP and ±0.13 mm ML, and the glass fiber was 0.175 mm long.

### Optogenetic stimulation protocol

DG engram cells were optogenetically stimulated with 15-ms pulses of 20 Hz (473 nm) by means of a laser (CrystaLaser), which was driven by a Transistor-Transistor Logic (TTL) input (Doric lenses). The power delivered at the DG was 10 to 15 mW (*16*, *43*). All optogenetic laser stimulation was conducted in the home cage of the test mouse, which was connected to the laser cables for 5 min.

### Drug preparation

#### Roflumilast

The drug preparation was performed as previously described (*16*). The PDE4 inhibitor roflumilast was dissolved in the vehicle solution containing 98% methyl cellulose Tylose and 2% Tween-80. All components were obtained from Sigma-Aldrich, Zwijndrecht, the Netherlands. Roflumilast was prepared fresh within 24 hours of the injection. The volume of the injection was calculated in proportion to the body weight, 2 ml/kg. Roflumilast was administered intraperitoneally at a dosage of 0.03 mg/kg, and Tylose without roflumilast served as a vehicle. Dose and injection volume were based on the previous work with roflumilast in memory studies (*16*). In the studies without light stimulation, the injections were either given immediately after socialization and 3 hours after socialization (Fig. 2, A to E) or 30 min before the social memory test (Fig. 2, F to O). For the study that combined pharmacological manipulations and engram tagging (Fig. 3), the injection was given 3 hours postoptogenetic stimulation. The timing was chosen as at this time, the inhibition of PDE4 is most effective for memory enhancement (*11*, *24*, *37*, *39*).

#### 4-Hydroxytamoxifen

4-Hydroxytamoxifen (4-OH-TAM; H6278, Sigma-Aldrich) was prepared as a stock solution at 50 mg/ml in dimethyl sulfoxide (DMSO, D8418, Sigma-Aldrich) and stored at −20°C. On the day of the experiment, a working solution of 4-OH-TAM (2.5 mg/ml) was prepared in two steps: First, the stock solution was diluted 1:10 in saline containing 2% Tween-80 (P1754, Sigma-Aldrich), followed by further dilution with saline. The final solution contained 4-OH-TAM (2.5 mg/ml), 5% DMSO, and 1% Tween-80 in saline. Mice received an intraperitoneal injection of 4-OH-TAM (25 mg/kg) 4 hours before the sample trials (*63*).

### Behavioral studies

#### Long-term SRM paradigm

Handling and habituation: Test mice were handled for 2 min every day for 3 to 5 days before the experiment to ensure that the mice are used to the experimenters (Fig. 1, A to E). A three-chambered arena was used to assess hippocampus-dependent long-term SRM. This task uses the innate preference toward a novel conspecific in favor of a familiar one and tests both social interaction and social recognition capacities of mice (*4*). The apparatus (60 cm by 40 cm by 20 cm) consisted of three chambers (20 cm by 40 cm by 20 cm). The chambers were separated with sliding door openings, and each side chamber contained a pencil cup. The habituation to the empty arena was performed at the beginning of the light phase (Zt0). The test mouse was placed in the central chamber of the empty arena and was allowed to explore it for 10 min. Then, the mouse was confined in the central chamber, and empty pencil cups were put in the side chambers. The mouse then was allowed to explore the arena with the pencil cups for 10 min. In addition, the littermates and the stimulus mice were habituated to the pencil cups for 2 min every day at least for 3 days before the experiment.

#### Socialization and social memory test

The paradigm has two key elements: socialization and social memory test (Fig. 1A). For socialization, a littermate and a stimulus mouse were placed in the pencil cups. The littermate was placed in one side chamber and the stimulus mouse in the other. The test mouse was placed in the central chamber of the arena with the doors to the side chambers closed. Then, the doors were opened, and the test mouse was given 10 min to explore the other mice. The positions of the littermate and the stimulus mouse within the arena were kept constant for each individual test mouse across the socialization and social memory test to avoid introducing spatial novelty as an additional variable. Across the cohort, however, the positions of the littermate and stimulus mouse were randomized and counterbalanced between the left and right chambers as well as between front and back of the arena. Thus, approximately, the same number of test mice were exposed to each of the littermate/stimulus location combinations. Between animals and trials, the pencil cups and the arenas were cleaned with 70% ethanol. For the social memory test, the littermate and the stimulus mouse were placed in the same location as during socialization. Again, the test mouse was allowed to explore the arena and the other mice for 10 min. The cleaning of the arena and the pencil cups was performed as previously described.

### Extended description of all behavioral training and testing protocols

#### Long-term social memory paradigm with SD

After establishing a working paradigm for long-term SRM, we examined the effects of SD on long-term SRM (Fig. 1, F to J). The protocol followed the steps as mentioned above. In addition, after the socialization session, the test subjects (housed together with their littermates) were sleep deprived for 6 hours with the gentle handling method.

#### Sleep deprivation

Immediately after the socialization session, the test mice were sleep deprived for 6 hours, except for the non–sleep-deprived groups (Fig. 1, F to J). SD was performed using the gentle handling method (*10*, *11*). It is aimed at causing minimal disturbance while keeping the animals awake by tapping on the cage, gently shaking the cage, and removing all the nesting material. The cage was shaken only when tapping was no longer sufficient. This SD method has been validated using the electroencephalography recordings, which indicated that it leads to the loss of all REM sleep and ∼95% NREM sleep (*64*). Previous studies have shown that SD by gentle handling does not lead to an increase in glucocorticoid levels as compared to the levels naturally occurring across the 24-hour cycle (*47*). Furthermore, we recently showed that while a mild increase in corticosterone occurs during SD, blocking glucocorticoid synthesis did not rescue the object-location memory impairment, indicating that stress hormone signaling is not the primary driver of hippocampal memory deficits in this context (*48*).

#### Long-term social memory paradigm with optogenetic manipulation

This experiment examined whether optogenetic stimulation can reverse the negative effects of SD on SRM (Figs. 1, K to P, and 3J). The test mice were naive to the experimental conditions and had never met the stimulus mice before. All mice were handled and habituated as described in the section “Long-term social memory paradigm” above. In addition, all the mice were scuffed on the last day of handling to habituate them to scuffing and habituated to the laser before the habituation to the arena. First, the test mouse was fixated on a sponge, and the laser cables were connected to the head implant. Then, the mouse was placed in its home cage for 5 min. The DG engram cells were optogenetically stimulated with 15-ms pulses of 20 Hz (473 nm, 10 to 15 mW) in accordance with previous work (*16*, *43*). After that, the cables were removed, and the mouse was subjected to the habituation to the empty arena. Of note, the test mice were kept on dox food (40 mg/kg) during habituation to prevent any tagging of neurons. Right after habituation, 24 hours before the socialization, the dox diet was replaced with normal chow. The next day, the test mice formed a social memory during the 10 min of socialization. The engram of the socialization event was tagged because of the dox food replacement. To prevent any further tagging, immediately after the socialization the test mice were put on a high-concentration dox diet (1000 mg/kg). The test mice were sleep deprived for 6 hours immediately following socialization. 24 hours after the socialization, the first memory test was performed. Right before the memory test, the test mice were connected to the laser cables and were placed in their home cage for 5 min. There, half of the animals received laser stimulation while the other half did not receive laser light. Next, the cables were removed, and the mice were subjected to the social memory test after 5 to 10 min. They were placed in the arenas with the littermates and the same stimulus mice from the socialization for 10 min. After the first memory test, the animals were left undisturbed for 5 days. Then, the second memory test was performed in the same manner as for the first memory test. However, the group that received laser stimulation before the first test was not exposed to the laser light before the second test, and vice versa.

#### Long-term social memory paradigm without functional ChR2

This experiment examined whether restoration of SRM depends on functional optogenetic activation of DG engram cells (Fig. 1, Q to V). The test mice were naïve to the experimental conditions and had never met the stimulus mice before. All mice were handled and habituated as described in the section “Long-term social memory paradigm” above. In addition, all mice were scuffed on the last day of handling to habituate them to scuffing and habituated to the laser before arena habituation. First, the test mouse was fixated on a sponge, and the laser cables were connected to the head implant. Then, the mouse was placed in its home cage for 5 min. Laser stimulation was delivered using the same parameters as in the optogenetic experiment (15-ms pulses at 20 Hz, 473 nm, 10 to 15 mW). All mice received laser stimulation; however, only half of the mice expressed functional ChR2 in the DG, while the remaining mice did not express ChR2. After this, the cables were removed and the mouse was subjected to habituation to the empty arena. Of note, the test mice were kept on dox food (40 mg/kg) during habituation to prevent any tagging of neurons. Immediately after habituation, 24 hours before socialization, the dox diet was replaced with normal chow. The next day, the test mice formed a social memory during the 10-min socialization session. Neuronal ensembles activated during socialization were tagged because of dox removal. To prevent any further tagging, immediately after socialization the test mice were placed back on a high-concentration dox diet (1000 mg/kg). The test mice were sleep deprived for 6 hours immediately following socialization. Twenty-four hours after socialization, the social memory test was performed. Right before the memory test, the test mice were connected to the laser cables and placed in their home cage for 5 min. All mice received laser stimulation. Next, the cables were removed, and the mice were subjected to the social memory test after 5 to 10 min. They were placed in the arenas with the cagemate and the same stimulus mouse from the socialization session for 10 min.

#### Long-term social memory paradigm with roflumilast

All mice were handled and habituated as described in the section “Long-term social memory paradigm” above. In addition, the test mice were given a mock intraperitoneal injection, i.e., insertion of the needle without a solution on the last day of handling and weighed to determine the dosage of the solution. Test mice were randomly divided into two groups: a sleep-deprived group and non–sleep-deprived group. The test mice in the sleep-deprived group were sleep deprived for 6 hours immediately after the socialization session. After 6 hours, animals were left to rest until the social memory test. The non–sleep-deprived group was left undisturbed after the socialization session until the start of the social memory test. For experiments assessing the effect of roflumilast on memory consolidation (Fig. 2, A to E), test mice received an intraperitoneal injection of either roflumilast or vehicle solution immediately after the socialization session and again 3 hours later. For experiments assessing the effect of roflumilast on memory retrieval, the test mice were injected with either roflumilast or a vehicle solution 30 min before the social memory test. The social memory test was performed 1 day (Fig. 2, F to J) or 5 days (Fig. 2, K to O) after the socialization, as described in the section “Long-term social memory paradigm.”

#### Long-term social memory paradigm with optogenetic and pharmacological manipulations

This experiment tested the capability of optogenetic and pharmacological stimulation to restore the SRM impaired by SD and to make the memory accessible in the long-term (Fig. 3, A to E). All mice were handled and habituated as described in the section “Long-term social memory paradigm” above. In addition, all the mice were habituated to scuffing and to the laser. This was performed as described in the section “Long-term social memory paradigm with optogenetic manipulation” above. The test mice were also given a mock intraperitoneal injection, i.e., insertion of the needle without a solution. Furthermore, they were weighed to determine the dosage of the solution. Twenty-four hours before the socialization, the test mice were taken off dox food. During the socialization, the test mice were allowed to explore the littermate and a stimulus mouse for 10 min. After socialization, the diet was immediately changed to high-concentration dox food (1000 mg/kg), and the mice were subjected to 6 hours of SD. For the next 3 days, the mice were left undisturbed. On the 3rd day after socialization, the mice were subjected to memory reactivation, and the mice were divided into three experimental groups: (i) optogenetics, (ii) roflumilast, and (iii) optogenetics + roflumilast. The optogenetics only group was exposed to the laser light and injection of a vehicle. The roflumilast group was injected with a PDE4 inhibitor roflumilast and was not exposed to the laser light. The optogenetics + roflumilast group was exposed to the laser light and injected with roflumilast. The optogenetic reactivation was done in the home cage, and the mice were connected to the laser cables for 5 min. The DG engram cells were optogenetically stimulated with 15-ms pulses of 20 Hz (473 nm, 10 to 15 mW) as in previous work (*16*, *43*). Three hours after the optogenetic reactivation, the mice were injected with either roflumilast or vehicle intraperitoneally; 2 days later the social memory test took place, with the same littermate and stimulus mouse from socialization. There was no laser exposure nor drug administration immediately before this memory test.

#### Long-term social memory paradigm with experience tagging

We examined whether optogenetic engram activation exclusively reengages the memory that has been tagged under SD conditions. All mice were handled and habituated to the arena, pencil cups, and optogenetic cables as described in the section “Long-term social memory paradigm” above.

During the first socialization session, the test mice were exposed to their cagemate and a stimulus mouse for 10 min, followed by 6 hours of SD or left undisturbed (Fig. 4, A to G, and fig. S2). Two days later, the test mice underwent a second socialization. The same cagemate assumed the position previously occupied by the stimulus. A new stimulus mouse was placed at the location formerly occupied by the cagemate in the initial socialization session. After the second socialization, the test mice again underwent 6 hours of SD or were left undisturbed. On the 6th day of the experiment, the test mice underwent a social memory test. They were presented with the stimulus mouse from the first socialization session and the stimulus mouse from the second session. For the social memory test, the position of each stimulus mouse was the same as in the socialization session.

For optogenetic tagging of specific contexts (Fig. 4, H to O), the experiments were conducted with two groups of test mice wherein both groups of mice were sleep deprived after each socialization session. In the first group, tagging occurred during the first socialization session. In the second group, tagging took place during the second socialization session. The animals that needed to be tagged were taken off dox 24 hours before the socialization sessions. Immediately after the socialization session, they were put back on high-concentration dox food. Last, both groups of test mice underwent laser stimulation 5 to 15 min before the social memory test as described in Long-term social memory paradigm with optogenetic manipulation.

#### Long-term social memory paradigm with dual engram labeling

We examined how SD affects the encoding and reactivation of neuronal ensembles associated with two consecutive social experiences within the same animal. All mice were handled and habituated to the arena and pencil cups as described in the section “Long-term social memory paradigm” above.

During the first socialization session, the test mice were exposed to their cagemate and a stimulus mouse for 10 min, followed by 6 hours of SD or left undisturbed. Two days later, the test mice underwent a second socialization session. The same cagemate assumed the position previously occupied by the stimulus mouse, while a new stimulus mouse was placed at the location formerly occupied by the cagemate in the initial socialization session. After the second socialization session, the test mice again underwent 6 hours of SD or were left undisturbed. On the 6th day of the experiment, the test mice underwent a social memory test, during which they were presented with the stimulus mouse from the first socialization session and the stimulus mouse from the second socialization session. For the social memory test, the position of each stimulus mouse was the same as during the corresponding socialization session. To independently label neuronal ensembles associated with the two social experiences (Fig. 4, P to W, and fig. S3, J and K), a dual activity–dependent tagging strategy was used. Tamoxifen was administered intraperitoneally 4 hours before the first socialization session to enable Cre-dependent EGFP labeling of neurons activated during this session. Dox was removed 24 hours before the second socialization session to permit tTA-dependent mCherry expression during the second social experience. Immediately after the second socialization session, the mice were returned to dox-containing food to prevent further tagging. No optogenetic stimulations was applied in this experiment.

### Pilot long-term social memory protocols

We attempted to use existing experimental protocols to assess SRM (*65*, *66*). However, these protocols failed to provide reliable outcomes for the assessment of SRM (fig. S1, A to J).

#### Pilot 1

The test subject (C57BL/6J-male) was habituated to the three-chamber apparatus for 10 min, during which it was allowed to explore the entire arena. After 10 min, the test subject was confined in the center chamber, while wire cups were placed in each of the side chambers. One cup contained an A/J-male, serving as stimulus, while the other cup remained empty. For socialization, the doors between chambers were opened, and the test subject explored the arena. Socialization lasted 10 min, and 24 hours later, the social memory test was performed. The stimulus mouse from the socialization was placed in the wire cup at the same location as before. In the other chamber, confined in a wire cup, a new stimulus mouse was placed. The test subject was again allowed to explore the arena, including the two stimulus mice, for 10 min. The time the animal spent exploring each wire cup was measured. If social memory was consolidated, it was expected that the test subject would spend more time exploring the novel mouse.

#### Pilot 2

The test subject (C57BL/6J-male) was habituated to the arena for 10 min in total. For the first 5 min of the habituation, the test subject was confined to the center chamber. For the last 5 min of the habituation, the test subject was allowed to explore the entire arena. After 24 hours, the test subject was introduced to two A/J-males in wire cups in opposite chambers. The socialization session lasted for 5 min. After 24 hours, the social memory of the test subject was assessed in a 5-min test. The test subject was placed in the arena containing one of the familiar mice from the previous day and one novel stimulus mouse (A/J-male). The time the animal spent exploring each wire cup was measured. If a consolidated memory of the familiar mouse was formed, it was expected that the test subject would spend more time exploring the novel mouse.

### Immunofluorescence

Immunofluorescence stainings was performed to visualize cFos and mCherry expression in hippocampal tissue sections from Figs. 1 and 3. Cups containing three to four dorsal brain sections were washed three times with 0.01 M phosphate-buffered saline (PBS) for 5 min each and then blocked with 0.3% H2O_2_ in PBS for 10 min. The sections were then incubated with 5% normal donkey serum (NDS) in PBST (0.2% Triton X-100) for 1 hour at room temperature to block nonspecific binding. Next, the sections were incubated with primary antibodies—mouse anti-cFos (1:1000, Abcam cFos) and rabbit anti-mCherry (1:1000, Invitrogen mCherry)—in PBS with 1% NDS and 0.2% Triton X-100 for three nights at 4°C. Following the primary antibody incubation, the sections were washed three times with PBS for 5 to 10 min each and then incubated with the secondary antibodies—donkey anti-cFos (1:400, the Jackson Laboratory) and donkey anti-mCherry (1:500, the Jackson Laboratory)—diluted in PBS with 1% NDS for 4 hours. After the secondary antibody incubation, the sections were washed four times with PBS for 5 min each. The sections were mounted on Superfrost microscope slides (Thermo Fisher Scientific) and coverslipped with Vectasheild Antifade mounting medium with DAPI (Vector Laboratories). Fluorescent signals were visualized using a Zeiss AXIO Observer microscope with 20× magnification. Images were captured using DAPI, mCherry, and cFos filters with exposures set to avoid overexposure while maintaining clear visibility of labeled cells. The slices were analyzed using Zeiss software to stitch and process images.

For the dual engram-labeling experiments (Fig. 4, P to W), immunofluorescence stainings were performed to visualize cFos, mCherry, and EGFP expression in hippocampal tissue sections. Mice were perfused, and brains were sectioned coronally at 20-μm thickness. For each animal, six dorsal DG sections were selected and processed together in individual netwells. Sections were first washed three times for 5 min in 0.01 M PBS and subsequently incubated in PBS containing 0.3% H_2_O_2_ and 20 mM glycine for 20 min to quench endogenous peroxidase activity and reduce background staining. Following this step, sections were washed three times for 10 min in PBS supplemented with 20 mM glycine. Nonspecific binding was blocked by incubating sections for 1 hour at room temperature in PBS containing 5% NDS, 0.2% Triton X-100, and 20 mM glycine. Sections were then incubated with primary antibodies diluted in PBS containing 1% NDS, 0.2% Triton X-100, and 20 mM glycine for three nights at 4°C. The following primary antibodies were used: mouse anti-cFos (1:1000; Abcam), rabbit anti-mCherry (1:1000; Invitrogen), and chicken anti-EGFP (1:1000; EMD Millipore). After primary antibody incubation, the sections were washed twice for 5 min and twice for 10 min in PBS containing 20 mM glycine. Secondary antibody incubation was performed for 4 hours at room temperature in the dark using donkey anti-mouse Alexa Fluor 568 (1:400), donkey anti-rabbit Alexa Fluor 647 (1:500), and donkey anti-chicken Alexa Fluor 488 (1:1000), diluted in PBS containing 1% NDS and 20 mM glycine. Sections were subsequently washed four times for 5 min in PBS containing 20 mM glycine. Sections were mounted onto Superfrost microscope slides using PBS, air-dried, and coverslipped with Vectashield antifade mounting medium containing DAPI (Vector Laboratories). Slides were stored at 4°C in the dark until imaging. Fluorescent signals were visualized using a Zeiss LSM 800 Airyscan confocal microscope with a 63× objective. Images were acquired using identical exposure settings across experimental groups for each fluorophore and processed using Zeiss software for stitching and analysis.

### Cell counting

To quantify the expression of ChR2-mCherry, EGFP, and cFos in the DG, the number of positive mCherry and cFos was assessed in three coronal slices per animal. Fluorescent images were captured using a Zeiss AXIO Observer microscope (Figs. 1 and 3) or using a Zeiss LSM 800 Airyscan confocal microscope (Fig. 4, P to W). Subsequently, Qupath software (v.0.4.3) was used for image analysis, with predesigned script (StarDist) optimized for automating cell counting (*67*). The cell body layer within the DG region was delineated as the region of interest. The StarDist script was then adapted to use DAPI staining for identifying total nuclei count. In addition, a classifier was trained within QuPath to identify and distinguish cFos-, EGFP-, and mCherry-positive cells. The model was trained across multiple images to accurately identify these cells within annotated regions. Subsequently, the trained classifier was applied to all slices to automate the counting of cFos-positive and mCherry-positive cells. The presence of cells positive for both cFos and mCherry was used to assess colocalization. Manual verification of cell counts was performed for each slice to rectify any errors, with counting conducted in a blinded manner to experimental conditions. The chance level of colocalization between cFos, EGFP, and mCherry was calculated depending on the experiment by determining the overall probability of a cell being positive for each marker. The probabilities were obtained by averaging the percentages of cFos and mCherry/EGFP-positive cells across all slices, or separately for the upper and lower blades of the DG. The chance level of colocalization was calculated as the product of these probabilities, expressed as a percentage.

### Behavioral scoring

The behavior in the socialization and memory test sessions was manually scored using the BORIS software (v. 7.13.9) (*68*). The researchers were blinded to the experimental group to which the test mouse belonged and to the positions of the littermate and the stimulus mouse in the cage. The time spent by the test mouse exploring the littermate and the stimulus mouse was recorded. The test mouse was considered to be exploring when it was directing the nose to the pencil cup at a distance of no more than 1 cm and/or touching the cup with the nose. On the basis of this time, the social preference index, a relative measure of discrimination between a familiar and an unfamiliar mouse, was calculated using the formula

Social preference index=(S−L)/(S+L)

where *S* is the time spent exploring the stimulus and *L* is the time spent exploring the littermate.

### Statistical analysis

Statistical analyses were performed and graphs were generated using GraphPad Prism 10 (GraphPad Software, La Jolla, CA). Data were tested for normality using the Shapiro-Wilk test. When applicable, homogeneity of variance was assessed before parametric testing. Exploration times toward the stimulus mouse and cagemate within the same session were analyzed using paired *t* tests when only two exploration targets were compared within animals (Figs. 1C and 4, B and D; and figs. S1, B and G, and S3, B and D). When exploration behavior was compared across experimental groups and targets, two-way analysis of variance (ANOVA) with repeated measures (within-subject factor: exploration target; between-subject factors: sleep condition, drug treatment, or viral condition) was used, followed by Bonferroni multiple comparison tests (Figs. 1, G, I, M, and O; 2, B, D, G, I, and N; 3, B and D; and 4, I, K, and N; and fig. S3, I, K, M, and O). Social preference indices were compared against chance level (0) using one-sample *t* tests (Figs. 1, E, H, J, N, and P; 2, C, E, H, J, and O; 3C, E, I, and M; and 4, C, E, G, J, L, and O; and figs. S1, E and J; S2, C, F, I, and L; and S3, C, E, G, L, N, and P). Comparisons between two independent groups were performed using two-tailed unpaired *t* tests for normally distributed data (Figs. 1, J and P, 3, J to M, and 4, J and L; and figs. S2, G to L, and S3H), or Mann-Whitney tests when normality assumptions were violated (Fig. 4S). For experiments involving more than two groups, one-way ANOVA was used when appropriate (Fig. 3, G and H, and fig. S2, A, B, D, and E), followed by Tukey’s post hoc tests. Nonnormally distributed datasets were analyzed using Kruskal-Wallis tests followed by Dunn’s multiple comparisons (Fig. 3I). Wilcoxon signed-rank tests were used for paired nonparametric comparisons where applicable (Fig. 4O). For the dual engram-labeling experiments, group differences in the proportion of EGFP-positive cells (Fig. 4S), mCherry-positive cells (Fig. 4T), EGFP + mCherry overlap (Fig. 4U), and cFos-positive cells (Fig. 4V) were assessed using unpaired *t* tests or Mann-Whitney tests as appropriate. Experience-specific engram reactivation, defined as overlap between cFos and each tagged ensemble, was analyzed using two-way ANOVA followed by Bonferroni post hoc comparisons (Fig. 4W). Results were considered statistically significant when *P* < 0.05. All data are presented as means ± SEM.
