# Supplementary material for: Restoring access to long-term social recognition memories disrupted by sleep deprivation
Source: Sci Adv. 2026 Jun 10;12(24):eadu9805. doi: 10.1126/sciadv.adu9805 (PMC13251821; doi:10.1126/sciadv.adu9805)
Supplement: Supplementary file 1 — Figs. S1 to S3 Tables S1 to S7 [file sciadv.adu9805_sm.pdf]

Science Advances
AAAS

Supplementary Materials for

Restoring access to long-term social recognition memories disrupted by sleep deprivation

Adithya Sarma *et al.*

Corresponding author: Robbert Havekes, r.havekes@rug.nl

Sci. Adv. **12**, eadu9805 (2026)
DOI: 10.1126/sciadv.adu9805

This PDF file includes:

Figs. S1 to S3
Tables S1 to S7

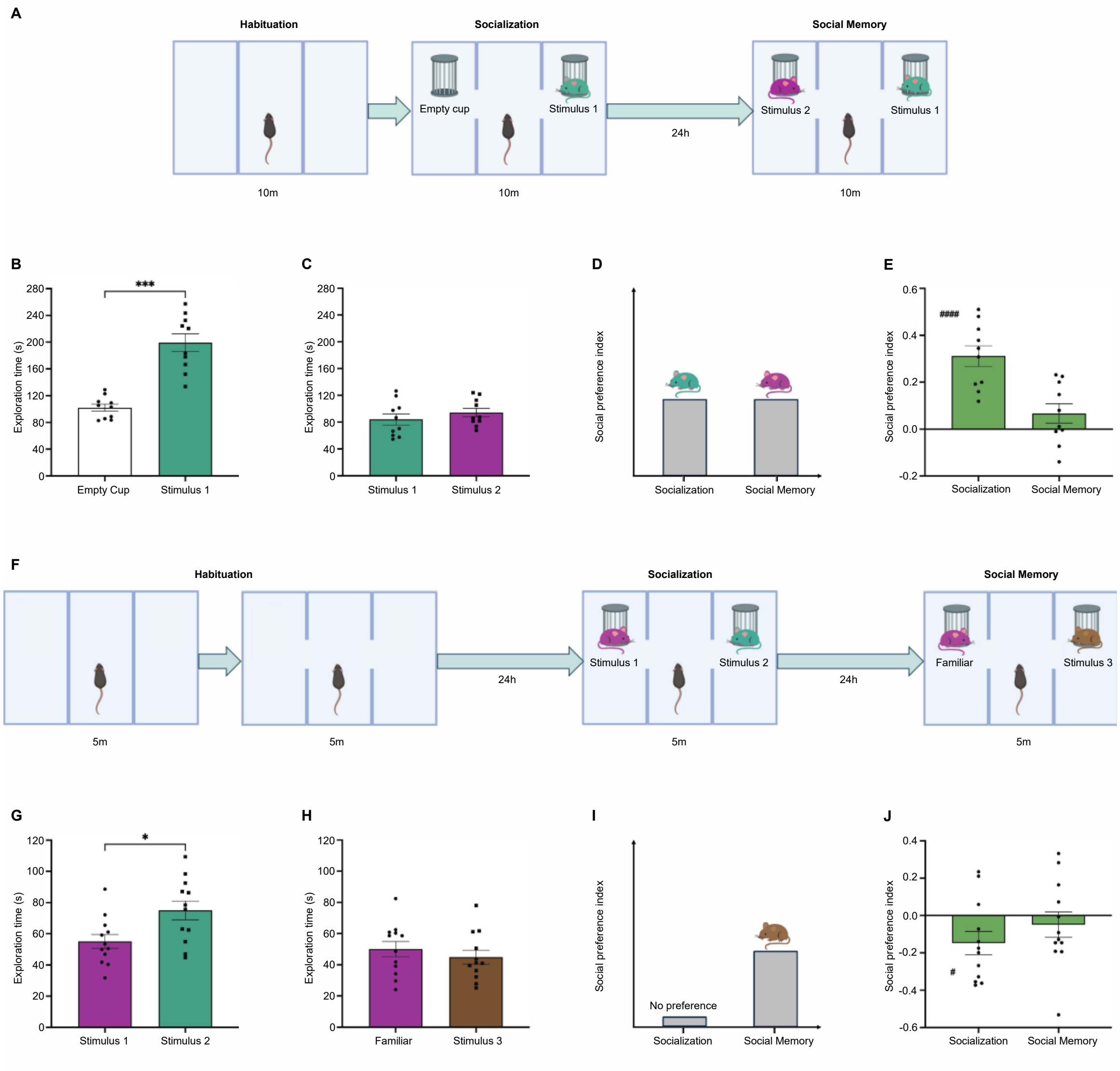

Figure S1.

Overview of the behavioral tests used to establish the paradigm for long-term social recognition memory.

(A) Test mice were habituated to the empty arena, followed by socialization in the arena with one empty wire cup and another wire cup with a novel stimulus mouse, placed in opposing chambers. After 24 h, they were exposed to the same stimulus mouse and a novel stimulus mouse to assess their social memory. **(B)** During the socialization session, test mice spent significantly more time exploring the novel stimulus mouse compared to the empty cup [n = 10, paired t-test, $t_9 = 6.159$, p = 0.0002]. **(C)** In the social memory test, test mice explored both the mice equally. **(D)** Expected outcome: during socialization, test mice were predicted to explore the stimulus mouse more than the empty cup. This would be reflected by a significantly positive social preference index. During the social memory test, if a consolidated memory was formed, the stimulus from the socialization would no longer be novel. Thus, the mice were expected to show a strong preference for the new

stimulus mouse over the familiar stimulus, which would be reflected by a significantly positive social preference index. **(E)** A positive social preference index significantly different from zero during the socialization session revealed a preference for the novel stimulus mouse over the empty cup [n = 10, one-sample t-test, t_9 = , $p < 0.0001$]. During the social memory test, no significant preference was observed for the novel stimulus mouse over the familiar stimulus [one-sample t-test, $t_9 = 1.622$, N.S.] **(F)** Test mice were habituated to the center chamber and then to the entire empty arena. During socialization, which took place 24 h later, the test mice were exposed to two stimulus mice simultaneously. After 24 h, they were exposed to one familiar stimulus mouse and to a novel stimulus mouse to assess their social memory. **(G)** The test mice explored one stimulus more than the other during socialization. Given that the numeration of stimulus mice was randomized, this indicated a chance preference to a random unfamiliar animal [n = 12, paired t-test, $t_{11} = 2.355$, $p = 0.0381$]. **(H)** In the social memory test, test mice explored both the mice equally. **(I)** Expected outcome: during socialization, test mice were predicted to explore both the stimulus mice equally, reflected by a social preference index close to zero. During the social memory test, if a consolidated memory was formed, the stimulus from the socialization would no longer be novel. Thus, the mice were expected to show a strong preference for the new stimulus mouse over the familiar stimulus mouse, which would be reflected by a significantly positive social preference index. **(J)** A negative social preference index significantly different from zero during the socialization session revealed a random preference for one novel stimulus mouse over the other one [n = 12, one-sample t-test, $t_{11} = 2.36$, $p = 0.0378$]. During the social memory test, test mice had no preference for either the familiar or the novel stimulus mouse [one-sample t-test, $t_{11} = 0.7075$, N.S.]. For detailed statistics, refer to Table S5. Sarma, A. (2026). Created using Biorender and Adobe Illustrator https://BioRender.com/27kudqe

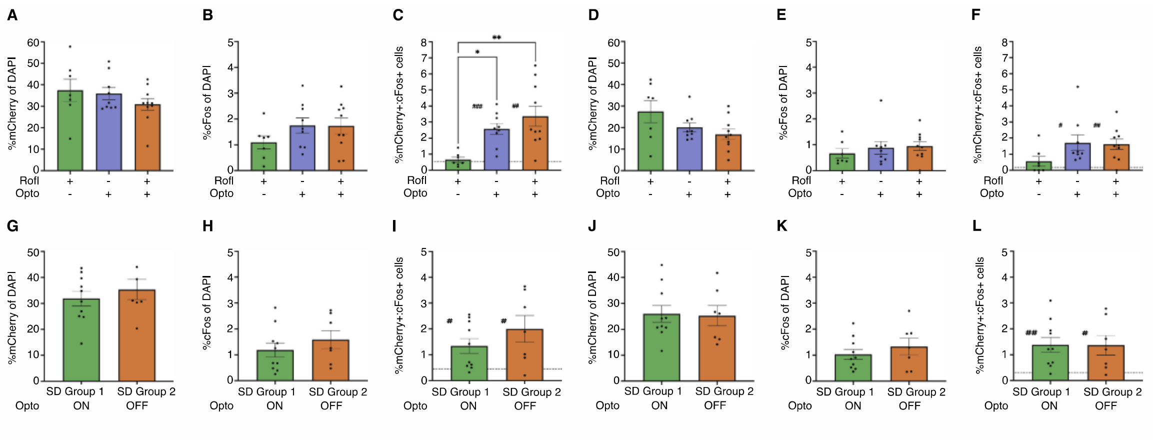

Figure S2.

Number of mCherry positive, c-Fos positive, or mCherry+c-Fos positive cells depicted as the percentage of the total number of DG cells, analyzed separately for the superior and inferior blades.

(A, B) No significant differences were detected in the percentages of mCherry-positive or c-Fos-positive cells in the DG superior blade among the three experimental groups [n = 7-10 per group, one-way ANOVA, N.S.]. **(C)** In the DG superior blade, colocalization levels did not exceed chance in the group that received only roflumilast treatment [chance level = 0.529, one-sample t-test, t_6 = 0.8555, N.S.]. However, colocalization significantly exceeded chance in mice subjected to optogenetic stimulation alone or in combination with roflumilast, indicating increased engram reactivation in these groups [chance level = 0.529, one-sample t-test, Optogenetics-only: t_8 = 5.99, p = 0.0003; Optogenetics and Roflumilast: t_9 = 4.545, p = 0.0014]. Additionally, colocalization was significantly lower in the roflumilast-only group compared to both the optogenetics-only and combination treatment groups [one-way ANOVA: $\mathbf{F}_{2,23}$ = 7.945, p = 0.0024; post-hoc Tukey's test: Roflumilast-only vs Optogenetics-only, p = 0.03; Roflumilast-only vs Optogenetics and Roflumilast, p = 0.0018]. **(D, E)** Analysis of the DG inferior blade showed no significant differences in the percentages of mCherry-positive or c-Fos-positive cells across the three treatment groups [n = 7-10 per group, one-way ANOVA, N.S.]. **(F)** For the DG inferior blade, colocalization did not exceed chance levels in the roflumilast-only group [chance level = 0.179, one-sample t-test, t_6 = 1.25, N.S.]. In contrast, colocalization was significantly above chance in mice that received optogenetic stimulation alone or in combination with roflumilast, indicating increased engram reactivation [chance level = 0.179, one-sample t-test, Optogenetics-only: t_8 = 3.144, p = 0.0137; Optogenetics and Roflumilast: t_9 = 4.444, p = 0.0016]. Although there were no significant group differences in colocalization within the inferior blade, a trend toward lower colocalization was observed in the roflumilast-only group compared to the other two groups [Kruskal-Wallis test: $\mathbf{H}_2$ = 5.957, p = 0.0509]. **(G, H)** In the superior blade of the DG, no significant group differences were observed in the percentages of mCherry-positive or c-Fos-positive cells across treatment groups [n = 7-10 per group, two-tailed unpaired t-test, N.S.]. **(I)** Laser stimulation delivered either one or six days post-socialization led to mCherry and c-Fos colocalization in the DG superior blade that significantly exceeded chance level when assessed six days post-socialization [chance level = 0.45, one-sample t-test, Group 1: t_9 = 3.119, p = 0.0123; Group 2: t_6 = 3.001, p = 0.024]. **(J, K)** In the inferior blade of the DG, no significant differences were identified in the percentages of mCherry-positive or c-Fos-positive cells across treatment groups [n = 7-10 per group, two-tailed unpaired t-test, N.S.]. **(L)** For the DG inferior blade, laser

stimulation administered one or six days post-socialization resulted in colocalization levels that significantly exceeded chance level when assessed six days post-socialization [chance level = 0.3, one-sample t-test, Group 1: $t_9 = 3.791$, $p = 0.0043$; Group 2: $t_6 = 2.796$, $p = 0.0313$]. Data are presented as mean ± SEM. Statistical significance is denoted as */#$p < 0.05$, **/##$p < 0.01$, ###$p < 0.001$. For detailed statistics, refer to Table S6.

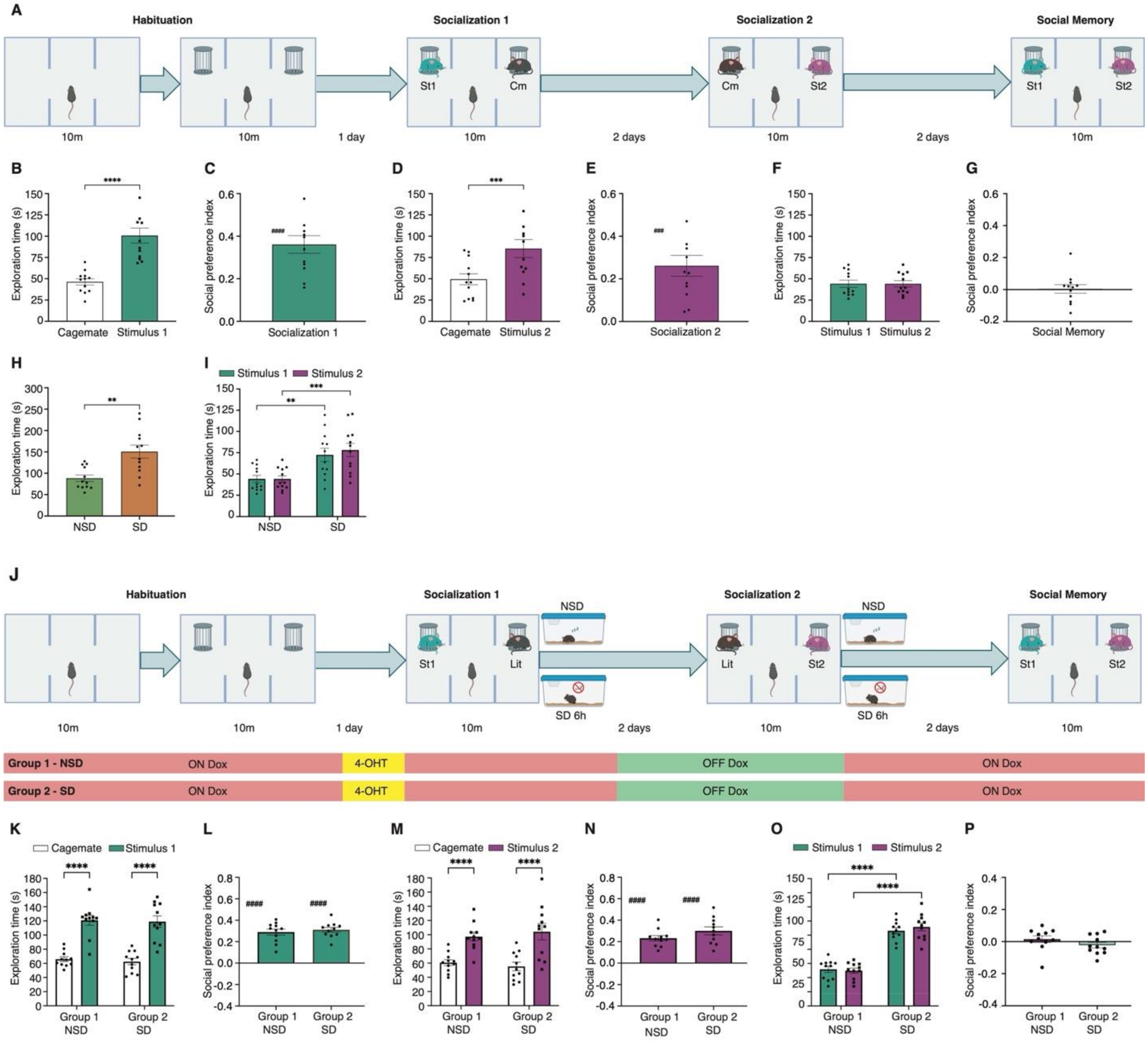

Figure S3.

Two social memories are formed in two consecutive socialization sessions and retrieved successfully under non-sleep deprived conditions.

(A) For this experiment, cagemates of test mice, with whom the test mice were pair-housed from the age of 6 weeks, were used instead of littermates. Test mice were habituated to the empty arena, followed by exposure to the arena with two empty wire cups in opposing chambers. After 24 h, test mice were exposed to their cagemate and a stimulus mouse. Two days later, the test mice were exposed to the same cagemate, now placed in the location previously occupied by the stimulus mouse, while a new stimulus mouse was placed where the cagemate had been. 6 days after the first socialization, the social memory test was conducted, with the stimulus mice from both sessions

presented in their original positions. **(B)** Test mice explored stimulus mice 1 more than the cagemate during the first socialization session [n = 12, paired t-test, t_{11} = 6.631, p < 0.0001]**. (C)** During the socialization session, a significant preference for the stimulus mouse was observed [one-sample t-test, t_{11} = 8.754, p < 0.0001]. **(D)** Test mice explored the stimulus mice more than the cagemate during the second socialization session [n = 12, paired t-test, t_{11} = 5.061, p = 0.0004]**. (E)** During the second socialization session, a significant preference for the stimulus mouse was observed [one-sample t-test, t_{11} = 5.310, p = 0.0002]. **(F)** Test mice explored both the stimulus mice equally during the social memory test [n = 12, paired t-test, t_{11} = 0.0357, N.S.]. **(G)** During the social memory test, no preference for either stimulus mouse was observed [one-sample t-test, t_{11} = 0.1546, N.S.]. **(H)** The total exploration time was significantly more for the sleep-deprived group than for the non-sleep-deprived group [n = 12 per group, two-tailed t-test, t_{22} = 0.3.667, p = 0.0014]. **(I)** Notably, the non-sleep-deprived mice explored both stimulus mice significantly less than their sleep-deprived counterparts [n = 12 per group, two-way ANOVA with repeated measures; significant main effect of sleep deprivation condition: $F_{1, 22}$ = 13.45, p = 0.0014, exploration time of stimulus 1: NSD vs SD, mean difference = -28.25 seconds, p < 0.0022, exploration time of stimulus 2: NSD vs SD, mean difference = -33.95 seconds, p = 0.0003]. **(J)** Experimental design showing two groups of test mice subjected to the two-session socialization paradigm, with one group allowed to sleep normally after each socialization session (NSD) and the other subjected to 6 hours of sleep deprivation (SD) following each session. Tamoxifen was administered intraperitoneally 4 hours before the first socialization session to enable Cre-dependent EGFP tagging, and doxycycline was removed 24 hours before the second socialization session to allow tTA-dependent mCherry tagging. **(K)** During the first socialization session, both NSD and SD mice explored stimulus mouse 1 significantly more than the cagemate [n = 11 per group, two-way ANOVA with repeated measures; no significant interaction between sleep condition and exploration target: N.S.; significant main effect of exploration target: $F_{1,20}$ = 182.9, p < 0.0001]. **(L)** A significant preference for stimulus mouse 1 was observed in both NSD and SD mice during the first socialization session [one-sample t-test; NSD: p < 0.0001; SD: p < 0.0001]. **(M)** During the second socialization session, both NSD and SD mice also explored stimulus mouse 2 significantly more than the cagemate [n = 11 per group, two-way ANOVA with repeated measures; no significant interaction between sleep condition and exploration target: N.S.; significant main effect of exploration target: $F_{1,20}$ = 80.24, p < 0.0001]. **(N)** A significant preference for stimulus mouse 2 was observed in both NSD and SD mice during the second socialization session [one-sample t-test; NSD: p < 0.0001; SD: p < 0.0001]. **(O)** During the social memory test, NSD mice explored stimulus mice 1 and 2 equally, whereas SD mice showed increased exploration of both stimulus mice [n = 11 per group, two-way ANOVA with repeated measures; significant main effect for sleep condition: $F_{1,20}$ = 95.94, p < 0.0001]. **(P)** No social preference for either stimulus mouse was observed in NSD mice, whereas SD mice showed elevated but non-selective exploration of both stimulus mice [one-sample t-test, NSD: N.S.; SD: N.S.]. Data are presented as mean ± SEM. Statistical significance is denoted as */#p < 0.05, **/##p < 0.01, ***/###p < 0.001, ****/####p < 0.0001. For detailed statistics, refer to Table S7. Sarma, A. (2026). Created using Biorender and Adobe Illustrator https://BioRender.com/axm8shi

Table S1.
Table of statistics for Figure 1.

Figure	Description	Test	Results	Post-hoc Bonferroni correction	N (per group)
**1C**	Exploration time during socialization (novel stimulus vs. littermate)	Paired t-test	$t_{11} = 5.198$, $p = 0.0003$		12
**1D**	Exploration time during social memory	Paired t-test	N.S.		12
**1E**	Social preference index	one-sample t-test	Socialization:<br>$t_{11} = 6.657$, $p < 0.0001$<br><br>Social Memory:<br>$t_{11} = 0.7429$, N.S.		12
**1G**	Exploration time during socialization	Two-way RM ANOVA	Condition x exploration target:<br>$F_{1,22} = 0.4569$, N.S<br><br>exploration target:<br>$F_{1, 22} = 78.78$, $p < 0.0001$	SD, NSD:<br>$p < 0.0001$	12
**1H**	Social preference index during socialization	One-sample t-test	NSD: $p < 0.0001$<br>SD: $p < 0.0001$		12
**1I**	Exploration time during social memory test	Two-way RM ANOVA	condition x exploration target:<br>$F_{1, 22} = 21.79$, $p < 0.0001$	SD:<br>$p < 0.0001$<br>NSD:<br>$p = 0.7195$	12
**1J**	Social preference index during social memory	one-sample t-test	NSD:<br>$t_{11} = 1.481$, $p = 0.1667$<br><br>SD:<br>$t_{11} = 5.232$, $p = 0.0003$		12
		Unpaired t-test	SD vs. NSD:<br>$t_{22} = 5.138$, $p = 0.0001$		12
**1M**	Exploration time during socialization	Two-way RM ANOVA	Group x exploration target:<br>$F_{1,17} = 0.02159$, N.S.<br><br>exploration target:<br>$F_{1, 17} = 39.52$, $p < 0.0001$	Group 1:<br>$p = 0.0007$<br>Group 2:<br>$p = 0.0007$	9-10

**1N**	Social preference index during socialization	One-sample t-test	Group 1: $p < 0.01$<br>Group 2: $p < 0.001$		9-10
**1O**	Exploration time during social memory test	Two-way RM ANOVA	Group (opto ON vs. OFF) x exploration target:<br>$F_{1,17} = 11.65$, $p = 0.0033$	SD OFF:<br>$p = 0.0003$<br><br>SD ON:<br>$p = 0.99$	9-10
**1P**	Social preference index during social memory test	One-sample t-test	SD OFF:<br>$t_9 = 5.132$, $p = 0.0006$<br><br>SD ON:<br>$t_8 = 0.4541$, N.S.		9-10
		Unpaired t-test	SD ON vs SD OFF:<br>$t_{17} = 3.759$, $p = 0.0016$		9-10
**1S**	Exploration time during socialization	Two-way RM ANOVA	Condition x exploration target:<br>$F_{1,22} = 0.9862$, N.S.<br><br>Exploration target:<br>$F_{1, 22} = 149.6$, $p < 0.0001$	Group 1:<br>$p < 0.0001$<br><br>Group 2:<br>$p < 0.0001$	12
**1T**	Social preference index during socialization	One-sample t-test	Group 1, $p < 0.001$<br>Group 2, $p < 0.001$		12
**1U**	Exploration time during social memory	Two-way RM ANOVA	group (ChR2 + vs. -) x exploration target (stimulus vs. cagemate):<br>$F_{1,22} = 46.52$, $p < 0.01$	SD+ChR2:<br>$p = 0.71$<br><br>SD-:<br>$p < 0.0001$	12
**1V**	Social preference index during social memory	One-sample t-test	SD+ChR2:<br>$t = 2.109$, N.S.<br><br>SD (mCherry):<br>$t = 8.61$, $p < 0.001$		12
		Unpaired t-test	$t = 8.043$, $p < 0.0001$		

Table S2.
Table of statistics for Figure 2.

**Figure**	**Description**	**Test**	**Results**	**Post-hoc Bonferroni correction**	**n**
**2B**	Exploration time during socialization	Two-way RM ANOVA	Group (NSDv, NSDr, SDv, SDr) x exploration target:<br>$F_{3,44} = 0.1931$, N.S<br><br>exploration target:<br>$F_{1,44} = 169.2$, $p < 0.0001$	All $p < 0.0001$	12
**2C**	Social preference index during Socialization	One-sample t-test	All $p < 0.01$		12
**2D**	Exploration time for social memory test	Two-way RM ANOVA	Group x exploration target:<br>$F_{3,44} = 16.88$, $p < 0.0001$	SD vehicle:<br>$p < 0.0001$<br><br>SD rofl:<br>N.S.	12
**2E**	Social preference index for social memory test	One-sample t-test	SD veh:<br>$p < 0.001$<br><br>SD rofl:<br>N.S.		12
**2G**	Exploration time during socialization	Two-way RM ANOVA	SD condition x drug condition x exploration target:<br>$F_{3,42} = 0.215$, N.S.<br><br>Exploration target:<br>$F_{1, 42} = 55.31$, $p < 0.0001$	NSDv: $p = 0.007$<br>SDv: $p = 0.0022$<br>NSDr: $p = 0.0047$<br>SDr: $p = 0.0004$	10-12
**2H**	Social preference index during Socialization	One-sample t-test	NSDv: $p < 0.001$<br>SDv, NSDr, SDr: $p < 0.01$		10-12
		Two-way ANOVA	N.S.		10-12

**2I**	Exploration time for social memory test	Two-way RM ANOVA	Group x exploration target:<br>$F_{3,42} = 6.624$, $p = 0.0002$	SDv: $p = 0.0003$<br>NSDv, NSDr, SDr: N.S.	10-12
**2J**	Social preference index for social memory test	One-sample t-test	SDv:<br>$t_{10} = 7.090$, $p < 0.0001$<br><br>SDr:<br>$t_{11} = 0.9560$, N.S.<br><br>NSDv, NSDr: N.S.		10-12
		Two-way ANOVA	SD condition x drug condition:<br>$F_{1,43} = 11.24$, $p = 0.0017$	SDv vs NSDv, $p < 0.01$<br>SDv vs NSDr, $p < 0.01$<br>SDv vs SDr, $p < 0.01$	10-12
**2L**	Exploration time during socialization	Two-way RM ANOVA	SD condition x drug condition x exploration target:<br>$F_{3,44} = 0.01$, N.S.<br><br>exploration target:<br>$F_{1, 44} = 132.6$, $p < 0.0001$	All conditions:<br>$p < 0.0001$	12
**2M**	Social preference index during socialization	One-sample t-test	NSDv, SDv, NSDr, SDr:<br>$p < 0.0001$		12
**2N**	Exploration time during social memory	Two-way RM ANOVA	Group x exploration target:<br>$F_{3,44} = 12.52$, $p < 0.0001$	SDv: $p < 0.0001$<br>NSDv, NSDr, SDr: N.S.	12
**2O**	Social preference index during social memory	One-sample t-test	SDv: $t_{11} = 7.362$, $p < 0.0001$<br>SDr: $t_{11} = 0.3736$, N.S.<br>NSDv, NSDr: N.S.		12
		Two-way ANOVA	SD condition x drug condition:<br>$F_{1,44} = 10.86$, $p = 0.0019$	SDv vs NSDv:<br>$p < 0.0001$<br>SDv vs NSDr:<br>$p < 0.0001$<br>SDv vs SDr: $p < 0.001$	

Table S3.
Table of statistics for Figure 3.

Figure	Description	Test	Results	Post-hoc	n
**3B**	Exploration time during socialization	Two-way RM ANOVA	treatment (Roflumilast, Optogenetics, Roflumilast and Optogenetics) x exploration target: $F_{2,25} = 1.324$, N.S.<br><br>exploration target: $F_{1,25} = 98.08$, $p < 0.0001$	Bonferroni correction:<br>Rofl: $p = 0.0002$<br>Opto: $p < 0.0001$<br>Rofl+Opto: $p < 0.0001$	9-10
**3C**	Social preference index during Socialization	One-sample t-test	Rofl, Opto, Rofl+Opto: $p < 0.001$		9-10
**3D**	Exploration time during social memory test	Two-way RM ANOVA	treatment x exploration target: $F_{2,25} = 4.644$, $p = 0.0193$	Bonferroni correction:<br>Rofl: $p = 0.0174$<br>Opto, Rofl+Opto: N.S.	9-10
**3E**	Social preference index during social memory test	One-sample t-test	Rofl: $t_8 = 4.073$, $p = 0.00036$<br>Opto: $t_8 = 0.08$, N.S.<br>Rofl+Opto: $t_9 = 0.605$, N.S.		9-10
		One-way ANOVA	$F_{(2,25)} = 8.168$, $p = 0.0019$	Dunnetts:<br>Rofl vs Opto: $p < 0.01$<br><br>Rofl vs Rofl+Opto: $p < 0.01$	
**3G**	%mCherry	One-way ANOVA	N.S.		7-10
**3H**	%cFos	One-way ANOVA	N.S.		7-10
**3I**	Colocalization %	One-sample t-test	chance level = 0.324<br><br>Rofl: $t_6 = 1.603$, N.S.		7-10

			Opto: t_8 = 5.074, p = 0.001<br>Opto+Rofl: t_9 = 4.98, $p < 0.001$		
		Kruskal-Wallis test	**H**$_2$ = 10.77, p = 0.0046	Dunn's test:<br>Rofl vs Opto: p = 0.02<br>Rofl vs Rofl+Opto: p = 0.0064	
**3J**	Social preference index (opto ON vs OFF) during social memory test 6d post-socialization	One-sample t-test	Group 1 (ON) : t_9 = 0.2459, N.S.<br>Group 2 (OFF): t_8 = 0.8184, N.S.		9-10
		Unpaired t-test	Opto ON vs. OFF:<br>t_{17} = 0.4596, N.S.		
**3K**	%mCherry (6d post-socialization)	Two-tailed t-test	N.S.		7-10
**3L**	%cFos (6d post-socialization)	Two-tailed t-test	N.S.		7-10
**3M**	Colocalization % (6d post-socialization)	one-sample t-test	chance level = 0.37<br><br>Group 1 (ON):<br>t_9 = 3.794, p = 0.004<br><br>Group 2 (OFF):<br>t_6 = 3.078, p = 0.0217		7-10

Table S4.
Table of statistics for Figure 4.

Figure	Description	Test	Results	Post-hoc Bonferroni correction	n
**4B**	Exploration time during first socialization	Paired t-test	$t_{11} = 6.904$, $p < 0.0001$		12
**4C**	Social preference index during first socialization	One-sample t-test	$t_{11} = 7.434$, $p < 0.0001$		12
**4D**	Exploration time during second socialization	Paired t-test	$t_{11} = 6.523$, $p < 0.0001$		12
**4E**	Social preference index during second socialization	One-sample t-test	$t_{11} = 8.083$, $p < 0.0001$		12
**4F**	Exploration time (stimulus 1 vs 2) during social memory test	Paired t-test	$t_{11} = 1.78$, N.S.		12
**4G**	Social preference index during social memory test	One-sample t-test	N.S.		12
**4I**	Exploration time during first socialization	Two-way RM ANOVA	Group x exploration target:<br>$F_{1,22} = 0.3201$, N.S.<br><br>exploration target:<br>$F_{1, 22} = 50.18$, $p < 0.0001$	Group 1: $p < 0.0001$<br>Group 2: $p = 0.0003$	12
**4J**	Social preference index during	One-sample t-test	Group 1:<br>$t_{11} = 6.232$, $p < 0.0001$<br>Group 2:<br>$t_{11} = 4.289$, $p = 0.0013$		12

	first socialization				
**4K**	Exploration time during second socialization	Two-way RM ANOVA	Group x exploration target:<br>$F_{1,22} = 3.811$, N.S.<br><br>exploration target:<br>$F_{1, 22} = 78.16$, $p < 0.0001$	Group 1: $p = 0.0001$<br>Group 2: $p < 0.0001$	12
**4L**	Social preference index during second socialization	One-sample t-test	Group 1:<br>$t_{11} = 7.430$, $p < 0.0001$<br>Group 2:<br>$t_{11} = 6.893$, $p < 0.0001$		12
**4N**	Exploration time during social memory test	Two-way RM ANOVA	exploration target:<br>$F_{1, 22} = 46.54$, $p < 0.0001$	Group 1: $p < 0.0001$<br>Group 2: $p = 0.0005$	12
**4O**	Social preference index	Wilcoxon Signed Rank Test	Group 1: W = 76, $p = 0.001$<br>Group 2: W = 72, $p = 0.0024$		12
**4S**	% of EGFP+ cells in DG (first socialization)	Mann-Whitney	U = 51, N.S.		11
**4T**	% of mCherry+ cells in DG (second socialization)	unpaired t-test	t = 1.088, N.S.		11
**4U**	Colocalization between EGFP and mCherry	unpaired t-test	t = 1.044, N.S.		11
**4V**	% of c-Fos-positive cells during social memory test	unpaired t-test	t = 1.254, N.S.		11
**4W**	Colocalization between c-Fos and EGFP/mCherry	Wilcoxon Signedt Rank Test fortest	Chance level (pooled) EGFP+cFos+ = 0.14%;<br><br>mCherry+cFos+ = 0.48%.		11

		against chance	NSD EGFP+cFos+:<br>W = 66, $p = 0.0010$;<br><br>NSD mCherry+cFos+:<br>W = 66, $p = 0.0010$;<br><br>SD EGFP+cFos+:<br>W = 54, $p = 0.0127$;<br><br>SD mCherry+cFos+:<br>W = 52, $p = 0.0186$.		
		Two-way RM ANOVA	Condition:<br>$F_{1,20} = 14.12$, $p = 0.001$	EGFP+cfos+:<br>$p < 0.05$<br><br>mCherry+cfos+:<br>$p < 0.05$	

Table S5.
Table of statistics for Figure S1.

Figure	Description	Test	Results	n
**S1B**	Exploration time during socialization	Paired t-test	$t_9 = 6.159$, $p = 0.0002$	10
**S1C**	Exploration time social memory test	Paired t-test	N.S.	10
**S1E**	Social preference index	One-sample t-test	Socialization: $t_9 = 7.101$, $p < 0.0001$<br>Social memory: $t_9 = 1.622$, N.S.	10
**S1G**	Exploration time during socialization	Paired t-test	$t_{11} = 2.355$, $p = 0.0381$	12
**S1H**	Exploration time during social memory test	Paired t-test	N.S.	12
**S1J**	Social preference index	One-sample t-test	Socialization: $t_{11} = 2.36$, $p = 0.0378$<br>Social memory: $t_{11} = 0.7075$, N.S.	12

Table S6.
Table of statistics for Figure S2.

Figure	Description	Test	Results	n
**S2A**	% mCherry (DG sup)	One-way ANOVA	N.S.	7-10
**S2B**	% cFos (DG sup)	One-way ANOVA	N.S.	7-10
**S2C**	Colocalization (DG sup)	One-sample t-test	chance level = 0.529<br><br>Rofl: $t_6 = 0.8555$, N.S.<br>Opto: $t_8 = 5.99$, $p = 0.0003$<br>Opto+Rofl: $t_9 = 4.545$, $p = 0.0014$	7-10
		One-way ANOVA	$\mathbf{F}_{2,23} = 7.945$, $p = 0.0024$<br><br>post-hoc Tukey's test:<br>Rofl vs Opto: $p = 0.03$<br>Rofl vs Opto+Rofl: $p = 0.0018$	7-10
**S2D**	% mCherry (DG inf)	One-way ANOVA	N.S.	7-10
**S2E**	% cFos (DG inf)	One-way ANOVA	N.S.	7-10
**S2F**	Colocalization (DG inf)	One-sample t-test	chance level = 0.179<br><br>Rofl: $t_6 = 1.25$, N.S.<br>Opto: $t_8 = 3.144$, $p = 0.0137$<br>Opto+Rofl: $t_9 = 4.444$, $p = 0.0016$	7-10
		Kruskal-Wallis	$\mathbf{H}_2 = 5.957$, $p = 0.0509$	
**S2G**	% mCherry (DG sup, Opto ON vs OFF)	two-tailed unpaired t-test	N.S.	7-10
**S2H**	% cFos (DG sup, Opto ON vs OFF)	two-tailed unpaired t-test	N.S.	7-10
**S2I**	Colocalization (DG sup, Opto ON vs OFF) post-socialization	One-sample t-test	chance level = 0.45<br><br>Group 1 (ON): $t_9 = 3.119$, $p = 0.0123$<br>Group 2 (OFF): $t_6 = 3.001$, $p = 0.024$	7-10
**S2J**	% mCherry (DG inf, Opto ON vs OFF)	two-tailed unpaired t-test	N.S.	7-10
**S2K**	% cFos (DG inf, Opto ON vs OFF)	two-tailed unpaired t-test	N.S.	7-10

**S2L**	Colocalization (DG inf, Opto ON vs OFF) 1/6d post-socialization	One-sample t-test	chance level = 0.3<br><br>Group 1: $t_9 = 3.791$, $p = 0.0043$<br>Group 2: $t_6 = 2.796$, $p = 0.0313$	7-10

Table S7.
Table of statistics for Figure S3.

Figure	Description	Test	Results	n
**S3B**	Exploration time during first socialization	Paired t-test	$t_{11} = 6.631, p < 0.0001$	12
**S3C**	Social preference index during first socialization	One-sample t-test	$t_{11} = 8.754, p < 0.0001$	12
**S3D**	Exploration time during second socialization	Paired t-test	$t_{11} = 5.061, p = 0.0004$	12
**S3E**	Social preference index during second socialization	One-sample t-test	$t_{11} = 5.310, p = 0.0002$	12
**S3F**	Exploration time during social memory test	Paired t-test	$t_{11} = 0.0357$, N.S.	12
**S3G**	Social preference index during social memory test	One-sample t-test	$t_{11} = 0.1546$, N.S.	12
**S3H**	Total exploration time (SD vs NSD)	Two-tailed t-test	$t_{22} = 0.3667, p = 0.0014$	12
**S3I**	Exploration time (stimulus x SD condition)	Two-way RM ANOVA	SD condition: $F_{1, 22} = 13.45, p = 0.0014$<br>**NSD vs SD**<br>stimulus 1:<br>mean difference = -28.25 seconds, $p < 0.0022$<br>stimulus 2:<br>mean difference = -33.95 seconds, $p = 0.0003$	12
**S3K**	Exploration time during first socialization	Two-way RM ANOVA	SD condition x exploration target: N.S.<br><br>Exploration target:<br>$F_{1,20} = 182.9, p < 0.0001$	11
**S3L**	Social preference index during first socialization	One-sample t-test	NSD: $p < 0.0001$<br>SD: $p < 0.0001$	11

**S3M**	Exploration time during second socialization	Two-way RM ANOVA	SD condition x exploration target: N.S.<br><br>exploration target: $F_{1,20} = 80.24$, $p < 0.0001$	11
**S3N**	Social preference index during second socialization	One-sample t-test	NSD: $p < 0.0001$<br>SD: $p < 0.0001$	11
**S3O**	Exploration time during social memory test	Two-way RM ANOVA	SD condition:<br>$F_{1,20} = 95.94$, $p < 0.0001$	11
**S3P**	Social preference index during social memory test	One-sample t-test	NSD: N.S.<br>SD: N.S.	11
